# Supplementary material for: zAvatar-test—A functional precision model to personalize ovarian cancer treatments: Results from a co-clinical study
Source: Cell Rep Med. 2025 Dec 30;7(1):102530. doi: 10.1016/j.xcrm.2025.102530 (PMC12866172; doi:10.1016/j.xcrm.2025.102530)
Supplement: Document S1. Figures S1–S4 and Tables S1–S6 [file mmc1.pdf]

Cell Reports Medicine, Volume *7*

Supplemental information

zAvatar-test—A functional precision model to personalize ovarian cancer treatments: Results from a co-clinical study

Marta F. Estrada, Filipa Amorim, Filipa Ferreira da Silva, Cátia Rebelo de Almeida, Márcia Fontes, Ricardo Coelho, Sónia Ferreira, Rita Canas-Marques, Mireia Castillo-Martin, João Casanova, Maria de Lurdes Batarda, Elisa Yaniz-Galende, Audrey LeFormal, Ana Marreiros, Francis Jacob, Viola Heinzelmann-Schwarz, Alexandra Leary, Henrique Nabais, and Rita Fior

Figure S1

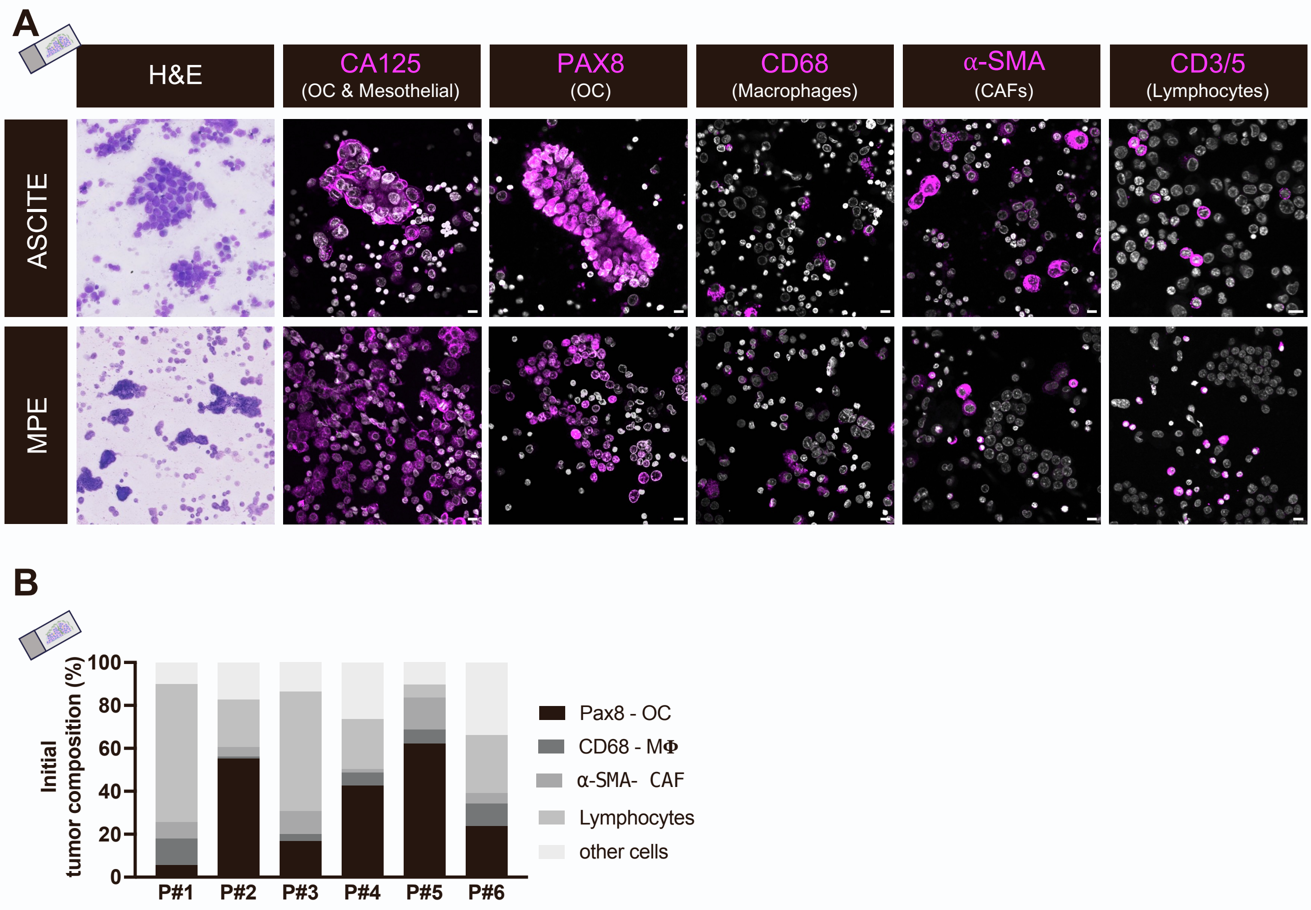

Figure S1. Comparison between samples obtained from different malignant effusions. Related to Figure 1. (A) Immunofluorescence characterization of patient cells before microinjection in smears, showing the presence of multiple cell types, including tumor cells (PAX8), tumor or mesothelial cells (CA125), macrophages (CD68), cancer-associated fibroblasts (a-SMA), lymphocytes (CD3/5), and erythrocytes (Giemsa staining). All immunofluorescence images are composites. **(B)** Analysis of cell composition in initial patient sample (smears) of the different cell populations.

Figure S2

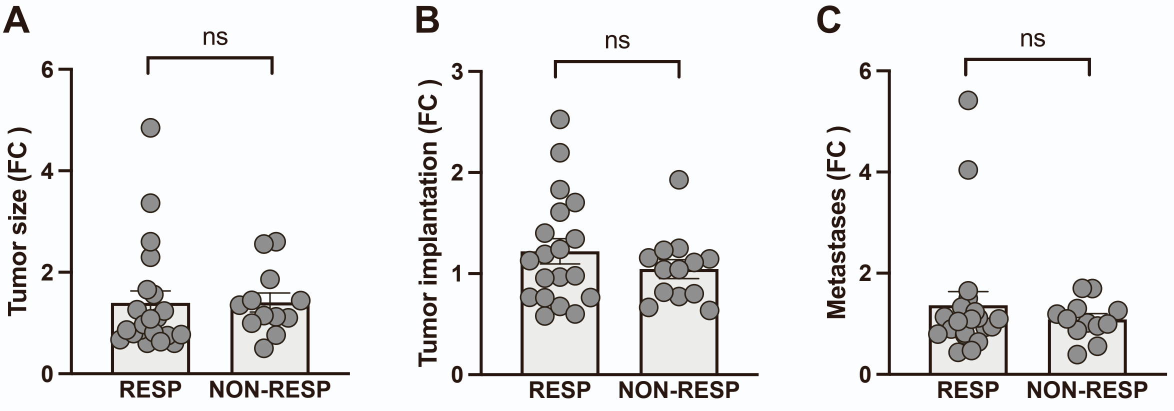

Figure S2. Tumor size, tumor implantation and micrometastases incidence in zAvatars in responder vs non-responder patients. Related to Figure 3. (A) Analysis of tumor size, **(B)** tumor implantation and **(C)** micrometastases incidence in zAvatars from responder vs non-responder patients. Data is expressed as AVG±SEM. Data were analyzed using unpaired two-sided Mann–Whitney test (ns > 0.05). Each dot represents one zAvatar. FC: fold change. RESP: responder. NON-RESP: non-responder.

Figure S3

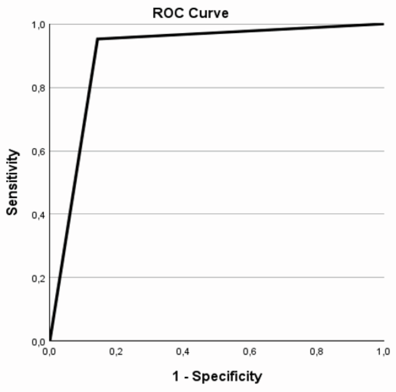

Figure S3. The zAvatar-test to discriminate responder from non-responder patients. Related to Figure 3. Receiver Operating Characteristic (ROC) curve analysis of the average fold change of apoptosis for both responder (N = 19 patients) and non-responder patients (N = 13 patients) of all 32 patients. The area under the curve was 0.905.

Figure S4

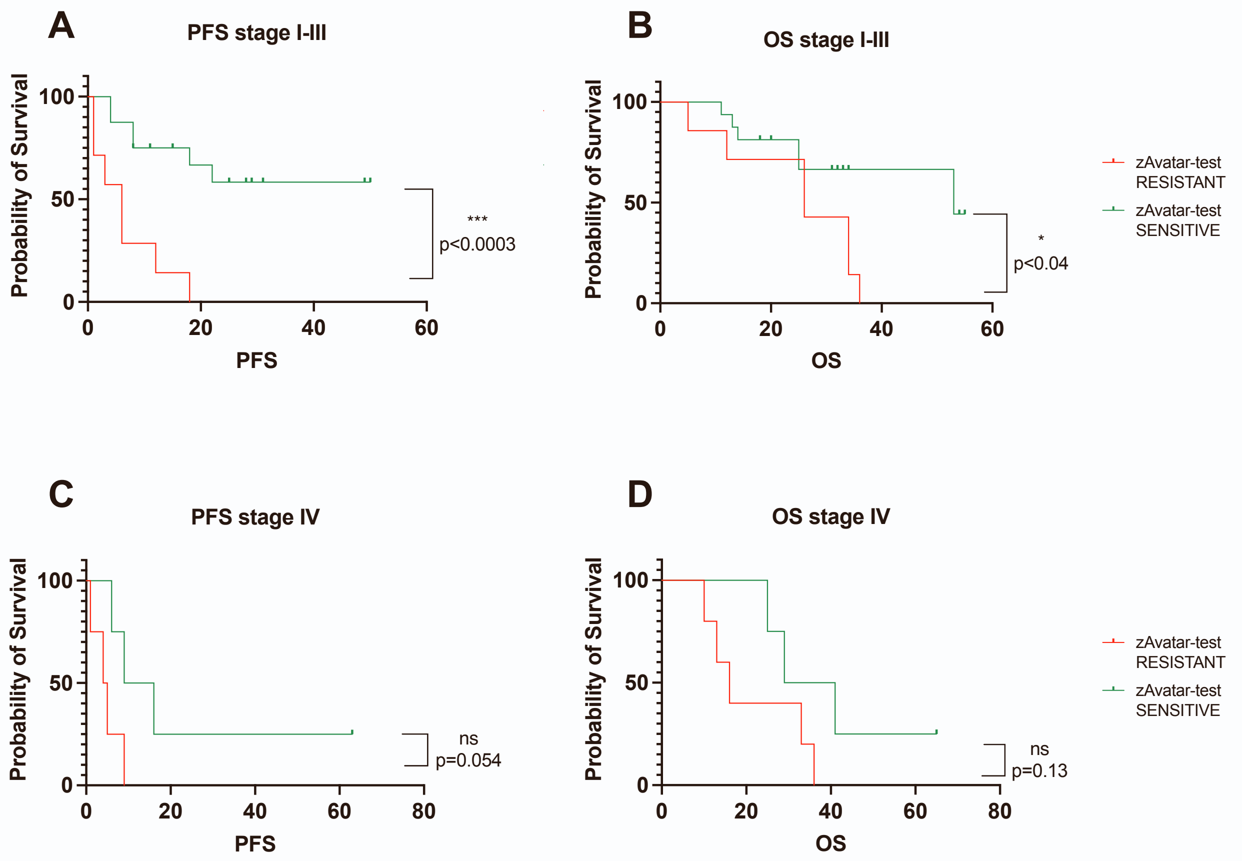

Figure S4. PFS and OS of patients with sensitive vs resistant zAvatar-test. Related to Figure 4. Kaplan–Meier survival curves to compare the PFS and OS of patients based on sensitivity or resistance of their zAvatar-test. Kaplan–Meier for PFS **(A, C)** and OS **(B, D)**. PFS: progression free survival. OS: overall survival.

Table S1. Study Population Baseline Characteristics. Related to Figure 1.

Study Population Baseline Characteristics (n=32)	
**Age at diagnosis - Years, average (s)**	59.4 (13.5)
**CA125 at sample collection - U/L, median (min-max)**	581 (30-15000)
**Histological types, n (%)**	
**Serous high grade**	28 (87.5%)
**Serous low grade**	2 (6.3%)
**Mucinous**	1 (3.1%)
**Clear Cell**	1 (3.1%)
**Neoadjuvant Chemotherapy, n (%)**	18 (56.3%)
**Primary surgery, n (%)**	29 (90.6%)
**FIGO staging, n (%)**	
**IA, n (%)**	2 (6.3%)
**IIIB, n (%)**	1 (3.1%)
**IIIC, n (%)**	20 (62.5%)
**IVA, n (%)**	4 (12.5%)
**IVB, n (%)**	5 (15.6%)
**BRCA mutation, n (%)**	2 (6.7%)
**Consolidation Chemotherapy, n (%)**	22 (68.8%)
**iPARP after consolidation CT, n (%)**	14 (43.8%)
**Bevacizumab after consolidation CT, n (%)**	5 (15.6%)
**Ascites at sample collection, n (%)**	16 (50%)
**Pleural effusion at sample collection, n (%)**	6 (18.8%)
**Ascites and pleural effusion at sample collection, n (%)**	1 (3.1%)

Table S2. Reagents for MIX1 preparation. Related to STAR Methods.

Reagents	Concentration	Vendor	Cat.no
Advanced DMEM/F12		ThermoFisher Scientific	12634010
Hepes	1%	ThermoFisher Scientific	15630106
MEM Non-Essential Amino Acids Solution	1x	ThermoFisher Scientific	1114005
B-27™ Supplement	1x	ThermoFisher Scientific	17504044
Putrescine dihydrochloride	10 µg/mL	Sigma-Aldrich	P5780
Nicotinamide	10 mM	Sigma-Aldrich	72340
N-acetylcysteine	1.25 mM	Sigma-Aldrich	A9165
Hydrocortisone	0.5 µg/mL	Sigma-Aldrich	H0888
glutaGRO Supplement	1x	Corning	MT25015CI
Insulin-Transferrin-Selenium	1% (v/v)	Corning	15383661
Y-27632	10 µM	Selleckchem	S6390
EGF	50 ng/mL	Peprotech	AF-100-15
Primocin	100 µg/mL	Invivogen	ant-pm-1
Universal Nuclease	37.5 U/mL	Pierce	88700

Table S3 – Treatments administered to zAvatars. Related to STAR Methods.

Scheme	Generic name	Cmax Patients (μM)	Concentration in zAvatar medium (μM)	Reference for Cmax Patients
**carboplatin + paclitaxel**	carboplatin	135	675	[S1]
	paclitaxel	4.27	0.34	[S1]
**carboplatin + paclitaxel + olaparib**	carboplatin	135	675	[S1]
	paclitaxel	4.27	0.34	[S1]
	plaparib	5	50	[S1]
**carboplatin + paclitaxel + niraparib**	carboplatin	135	675	[S1]
	paclitaxel	4.27	0.34	[S1]
	niraparib	0.00165	250	[S1]
**carboplatin + paclitaxel + bevacizumab**	carboplatin	135	675	[S1]
	paclitaxel	4.27	0.34	[S1]
	bevacizumab	0.6-0.9	1.68	[S2]
**carboplatin + gemcitabine**	carboplatin	135	675	[S1]
	gemcitabine	89.3	160	[S1]
**carboplatin + doxorubicin**	carboplatin	135	675	[S1]
	doxorubicin	6.73	1.2	[S1]
**paclitaxel**	paclitaxel	4.27	0.34	[S1]
**doxorubicin**	doxorubicin	6.73	1.2	[S1]
**topotecan**	topotecan	0.02	0.02	[S1]
**paclitaxel + bevacizumab**	paclitaxel	4.27	0.34	[S1]
	bevacizumab	0.6-0.9	1.68	[S2]
**doxorubicin + bevacizumab**	doxorubicin	6.73	1.2	[S1]
	bevacizumab	0.6-0.9	1.68	[S2]
**topotecan + bevacizumab**	topotecan	0.02	0.02	[S1]
	bevacizumab	0.6-0.9	1.68	[S2]
**venetoclax**	venetoclax	4.48	0.448	[S1]
**carboplatin + paclitaxel + venetoclax**	carboplatin	135	675	[S1]
	paclitaxel	4.27	0.34	[S1]
	venetoclax	4.48	0.448	[S1]

Table S4 – Composition of the Tricaine solution. Related to STAR Methods.

**Tricaine 25x**	**Quantity**
Tricaine powder	2 g
Reverse osmosis water	500 mL
1 M Tris (pH 9)	10 mL
Adjust to pH 7	250 µL

Table S5 – Composition of the blocking solution. Related to STAR Methods.

**Reagent**	**Quantity**
PBS (1x)	To a final volume of 50 mL
BSA	0.5 g
DMSO	500 µL
Triton 10% (w/v)	250 µL
Goat serum	750 µL

Table S6 – Antibodies used in immunofluorescent staining. Related to STAR Methods.

	Antibody	Supplier	Host animal	Dilution factor	Clones
**Primary antibodies**	Anti-Human mitochondria	Sigma Life Sciences	Mouse	1:50	113- 1
	Anti-Caspase 3	Cell Signaling Technologies	Rabbit	1:100	5A1E
	Anti-PAX8	Abcam	Rabbit	1:500	EPR18715
	Anti-CA125	Leica Biosystems	Mouse	Ready to use	OV185:1
	Anti-CD68	Leica Biosystems	Mouse	Ready to use	514H12
	Anti-aSMA	Abcam	Rabbit	1:2500	EPR5368
	Anti-CD3	Leica Biosystems	Mouse	Ready to use	CD3-565-L-CE
	Anti-CD5	Leica Biosystems	Mouse	Ready to use	CD5-4C7-L-CE
**Secondary antibodies**	Anti-mouse 488	ThermoFisher Scientific	Goat	1:400 (whole mount IF)<br>1:200 (smear)	
	Anti-mouse 594	ThermoFisher Scientific	Goat	1:400 (whole mount IF)<br>1:200 (smear)	-
	Anti-rabbit 488	ThermoFisher Scientific	Goat	1:400 (whole mount IF)<br>1:200 (smear)	-
	Anti-rabbit 594	ThermoFisher Scientific	Goat	1:400 (whole mount IF)<br>1:200 (smear)	-
	DAPI	Invitrogen	-	1:100 (whole mount IF)<br>15:10000 (smear)	-

Supplemental references

[S1] D.R. Liston, M. Davis, Clinically Relevant Concentrations of Anticancer Drugs: A Guide for Nonclinical Studies, Clin. Cancer Res. 23 (2017) 3489–3498. doi:10.1158/1078-0432.CCR-16-3083.

[S2] C. Rebelo de Almeida, R.V. Mendes, A. Pezzarossa, J. Gago, C. Carvalho, A. Alves, et al., Zebrafish xenografts as a fast screening platform for bevacizumab cancer therapy, Commun Biol. 3 (2020) 299–13. doi:10.1038/s42003-020-1015-0.
