# Supplementary material for: zAvatar-test—A functional precision model to personalize ovarian cancer treatments: Results from a co-clinical study
Source: Cell Rep Med. 2025 Dec 30;7(1):102530. doi: 10.1016/j.xcrm.2025.102530 (PMC12866172; doi:10.1016/j.xcrm.2025.102530)
Supplement: Document S2. Article plus supplemental information [file mmc3.pdf]

Cell Reports
Medicine

Article

zAvatar-test—A functional precision model to personalize ovarian cancer treatments: Results from a co-clinical study

Graphical abstract

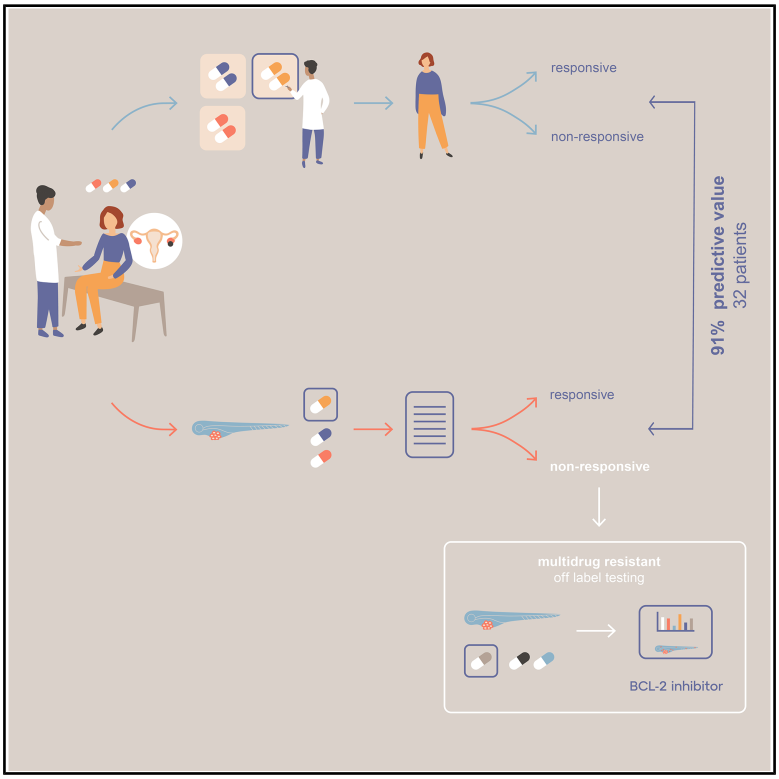

Highlights

- zAvatar-test predicts ovarian cancer therapy response with 91% accuracy (32 patients)
- zAvatar-sensitive patients show longer progression-free survival and overall survival
- Treatment efficacy tested in 10 days without *in vitro* cell expansion
- Ongoing randomized trial will evaluate the zAvatar-test's clinical benefit

Authors

Marta F. Estrada, Filipa Amorim, Filipa Ferreira da Silva, ..., Alexandra Leary, Henrique Nabais, Rita Fior

Correspondence

marta.estrada@research.fchampalimaud.org (M.F.E.), rita.fior@research.fchampalimaud.org (R.F.)

In brief

Estrada et al. report a co-clinical study showing that patient-derived ovarian cancer zebrafish Avatars (zAvatars) predict therapeutic response with 91% accuracy, in a cohort of 32 patients. The zAvatar-test stratifies patients by survival outcomes and enables drug repurposing, advancing functional precision oncology.

Estrada et al., 2026, Cell Reports Medicine 7, 102530
January 20, 2026 © 2025 The Author(s). Published by Elsevier Inc.
https://doi.org/10.1016/j.xcrm.2025.102530

Cell Reports Medicine

CellPress
OPEN ACCESS

Article

zAvatar-test—A functional precision model to personalize ovarian cancer treatments: Results from a co-clinical study

Marta F. Estrada,[1,*] Filipa Amorim,[1] Filipa Ferreira da Silva,[2] Cátia Rebelo de Almeida,[1] Márcia Fontes,[1] Ricardo Coelho,[3] Sónia Ferreira,[2] Rita Canas-Marques,[4] Mireia Castillo-Martin,[4] João Casanova,[2,5] Maria de Lurdes Batarda,[2] Elisa Yaniz-Galende,[6] Audrey LeFormal,[6] Ana Marreiros,[7,8,9] Francis Jacob,[3] Viola Heinzelmann-Schwarz,[3] Alexandra Leary,[6] Henrique Nabais,[2] and Rita Fior[1,10,*]

[1]Champalimaud Research, Champalimaud Foundation, 1400-038 Lisbon, Portugal
[2]Gynecology Department, Champalimaud Clinical Centre, Champalimaud Foundation, 1400-038 Lisbon, Portugal
[3]Department of Biomedicine, University Hospital and University of Basel, 4031 Basel, Switzerland
[4]Pathology Service, Champalimaud Clinical Centre, Champalimaud Foundation, 1400-038 Lisbon, Portugal
[5]Gynecologic Oncology Unit, Obstetrics and Gynecology Service, Department of Surgery, Hospital da Luz Lisboa, 1500-650 Lisbon, Portugal
[6]Department of Medical Oncology at Gustave Roussy, 94805 Paris, France
[7]Faculty of Medicine and Biomedical Sciences, University of Algarve, 8005-139 Faro, Portugal
[8]Algarve Biomedical Center Research Institute, University of Algarve, 8005-139 Faro, Portugal
[9]Unidade Local de Saúde do Algarve, 8000-386 Faro, Portugal
[10]Lead contact
*Correspondence: marta.estrada@research.fchampalimaud.org (M.F.E.), rita.fior@research.fchampalimaud.org (R.F.)
https://doi.org/10.1016/j.xcrm.2025.102530

SUMMARY

In ovarian cancer, 80% of patients relapse after first-line therapy. In recurrent cases, oncologists lack reliable tests to guide chemotherapy choices, creating an unmet clinical need. Here, we develop the ovarian cancer zebrafish Avatar-test, a functional *in vivo* model using patient tumor cells implanted in zebrafish embryos to predict treatment responses. We present the largest observational study (32 patients), where the zAvatar-test achieves 91% accuracy in predicting patient outcomes. Patients with a zAvatar-sensitive-test correlate with longer progression-free survival (17 vs. 6 months). Tumors in zAvatars are dynamic, with human-host cell interactions, and higher metastatic potential in poor-prognosis cases. Finally, as a proof of concept, we demonstrate that venetoclax has the potential to sensitize multidrug-resistant tumors. Altogether, this clinical study demonstrates that the zAvatar-test may help clinicians personalize treatments for ovarian cancer patients. We are now conducting a multicentric randomized clinical trial to evaluate the zAvatar-test as a companion tool in clinical oncology.

INTRODUCTION

Ovarian cancer (OC) is the most lethal gynecological cancer due to late diagnosis and drug resistance.[1] OC is a very heterogeneous disease with distinct molecular and histologic phenotypes. The most common and aggressive subtype is the high-grade serous histologic subtype, which accounts for 70%–80% of all OC-related deaths.[2] In most cases, patients are only diagnosed in advanced stages (III or IV), where metastatic spread has already occurred, decreasing drastically the 5- and 10-year survival rate to <30% and <15%, respectively.[3]

OC commonly disseminates within the peritoneal cavity—cells typically detach from the primary tumor and form implants in the peritoneal lining and in the omentum, leading to the accumulation of ascites in the abdominal cavity (~50% of patients). In more advanced stages, OC cells can also invade the pleura causing malignant pleural effusions (MPEs). These malignant effusions are composed by a variety of cellular and acellular components, including tumor cells, immune cells, erythrocytes, cancer-associated fibroblasts (CAFs), matrix components, cytokines, and growth factors. The presence of malignant effusions is typically associated with a poor prognosis, as they are known to facilitate metastases and contribute to chemoresistance.[4]

Initial therapy consists in a combination of cytoreductive surgery and platinum-based chemotherapy. Maintenance therapy with PARPi and/or bevacizumab is often utilized, according to homologous recombination deficiency and BRCA mutational status. In the setting of unresectable disease, or important comorbidities, neoadjuvant chemotherapy followed by interval cytoreductive surgery is an option for patients with advanced-stage OC.[5]

Despite the high response rates to platinum-based chemotherapy, most patients relapse (>80%) within 5 years.[6] At this stage, patients face a wide range of second-line chemotherapy options (6–8 options), with limited molecular markers available

 © 2025 The Author(s). Published by Elsevier Inc.
This is an open access article under the CC BY license (http://creativecommons.org/licenses/by/4.0/).

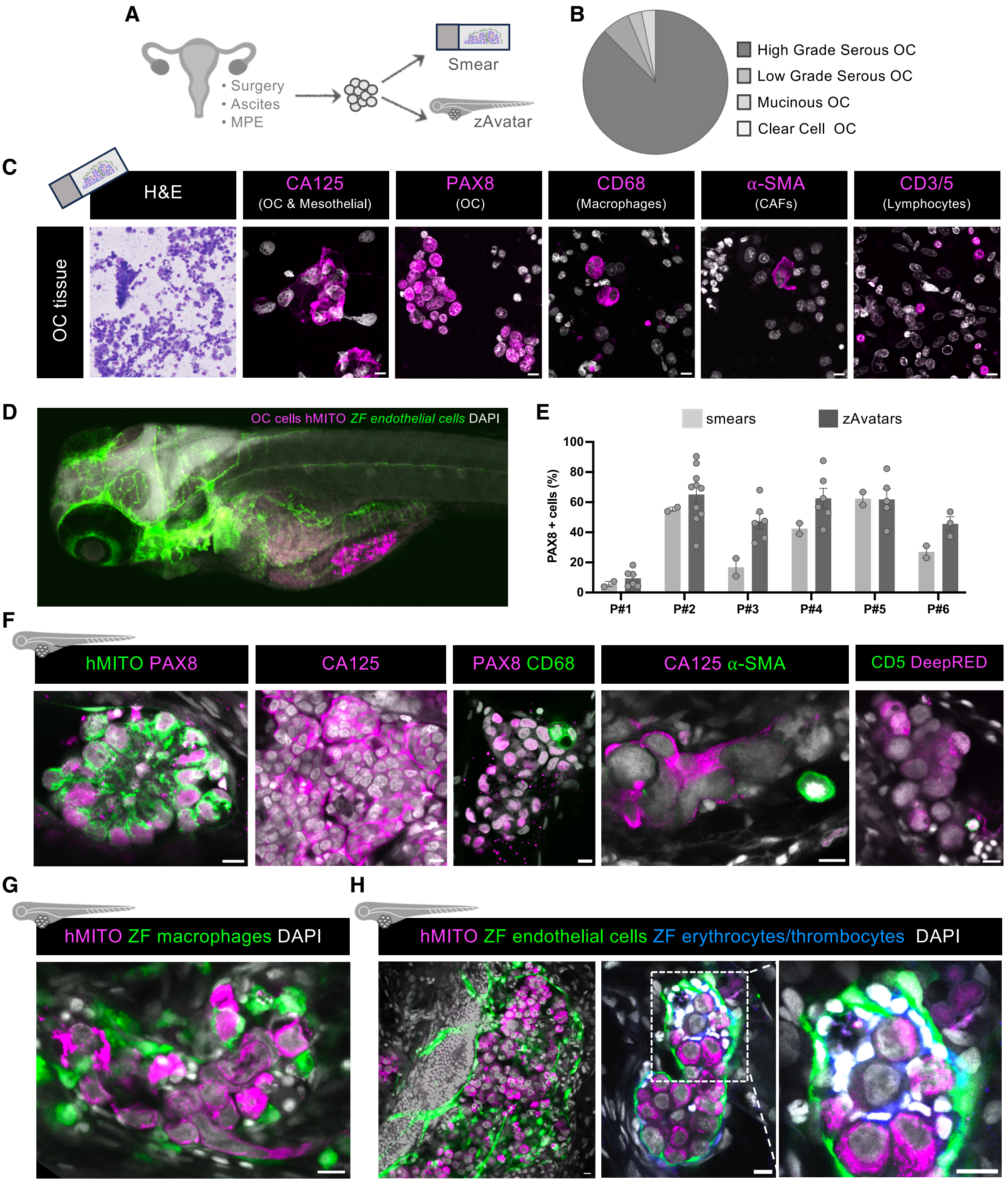

Figure 1. zAvatars preserve key features of the original patient tumor and create a dynamic TME

(A) Schematic illustration of the workflow for validation of the OC zAvatar model by comparing the cell composition of respective smear before microinjection with the zAvatar.

(B) Histological types of OC patients included in this study.

(C) Characterization of patient cells before microinjection in smears show the presence of multiple cell types, including erythrocytes (Giemsa staining), tumor or mesothelial cells (CA125), tumor cells (PAX8), macrophages (CD68), cancer-associated fibroblasts (a-SMA), and lymphocytes (CD3/5)

(D) Representative confocal images of an OC zAvatar.

(E) Percentage of PAX8-positive cells in the initial patient sample (smears) vs. tumors in zAvatars at 3 dpi. Results are expressed as AVG ± SEM. Each dot represents one smear and/or one zAvatar.

(legend continued on next page)

CellPress
OPEN ACCESS

to guide treatment decisions.[7] As a result, therapeutic options may result in ineffective treatments, which may promote clonal selection and immunosuppression, providing a competitive milieu for tumor progression and acquisition of multidrug-resistant states.[8]

Thus, the combination of patient stratification based on predictive biomarkers with direct challenging of live tumor cells is necessary to guide patient treatment and improve patient's survival outcomes.

We have developed a 10-day *in vivo* test with cellular resolution: the zebrafish-embryo xenograft model (zAvatar). The zAvatar model retains the complexity of an *in vivo* system, while offering significant advantages in terms of speed and resolution. This highly sensitive and dynamic assay captures tumor heterogeneity and allows the evaluation of therapeutic cytotoxicity in less than 10 days.[9,10] Moreover, their transparency enables whole-body imaging with single-cell resolution, where the metastatic potential, angiogenesis, and innate immune interactions[11,12] can be accessed in real time.

To assess the predictive value of the OC zAvatar model, we performed an observational study in which zAvatar treatments were aligned with the therapies received by the corresponding patients. Our results showed that OC zAvatars mimicked aspects of the initial tumor sample and were composed of several cell types, including tumor cells, CAFs, lymphocytes, and macrophages. Patient cells actively interacted with host zebrafish cells within the first hours of injection, creating a dynamic tumor microenvironment (TME). As these interactions occurred, OC cells had the ability to disseminate to distinct anatomic locations in zAvatars, with cells from patients with a worse initial prognosis exhibiting a more aggressive phenotype.

Importantly, we demonstrate that zAvatars predicted the patient clinical outcome in 91% of the cases and that patients with a zAvatar-sensitive test had a significantly longer progression-free survival (PFS) and overall survival (OS) compared to those with a zAvatar-resistant test.

Finally, as a proof of concept we tested the BCL2 inhibitor venetoclax (Vx) as an off-label option to treat multidrug-resistant tumors. Our data strongly validate the utility of zAvatars not only as a potential screening tool to optimize systemic therapies in OC within the guidelines but also as test to find off-label options to help patients with multidrug-resistant tumors.

RESULTS

zAvatars preserve key features of the original patient tumor and create a dynamic TME

To develop the OC zAvatars for personalized medicine, we collected samples derived from peritoneal implants (PI), ascites, or pleural effusions (MPEs) from 50 OC patients (Figure 1A; Table S1). Samples collected were from patients in the adjuvant setting and relapsed patients, the majority being high-grade serous ovarian carcinomas and also two low-grade ovarian carcinomas, one clear cell carcinoma, and one mucinous carcinoma (Table S1; Figure 1B).

All samples were prepared without *in vitro* expansion, followed by injection into 2 days post-fertilization (dpf) zebrafish embryos. zAvatars were successfully generated from 32 patients in a total of 50, with a success rate of 64%. Samples that failed were mostly from peritoneal tumor tissue samples.

To evaluate whether tumors in zAvatars were representative of the original patient tumor sample, before injection we prepared smears of the cell suspension to characterize and compare the initial cell composition with the corresponding zAvatars (Figures 1A, 1C–1E, and S1A). Our results from the smears show that OC patient samples were composed by several cells types: OC cells ($PAX8^+$)[13]; OC and mesothelial cells ($CA125^+$)[14]; CAFs (α-SMA)[15]; macrophages ($CD68^+$)[16]; and lymphocytes ($CD3/5^+$)[17] (Figure 1C). Quantification of the cell types present in each sample revealed a high interpatient heterogeneity, which is in line with what has been described in the literature (Figure S1B).[18] The content of lymphocytes was also highly variable, and that impacted on the survival of zAvatars. More specifically, we observed that if lymphocytes composed more than 60%–70% of the sample, most zAvatars died within the first 24 h post-injection. Conversely, if the content of PAX8-positive cells was lower than 10%, there was no tumor implantation.

In zAvatars, tumors were mostly composed by OC cells ($PAX8^+$ and $CA125^+$), and also of human macrophages ($CD68^+$), CAFs ($α\text{-}SMA^+$), and human lymphocytes ($CD5^+$) (Figure 1F), effectively recapitulating the original tumor composition from each patient. Additionally, quantification of PAX8-positive cells in zAvatars showed that their average percentage closely mirrored that of the initial patient sample (smears) (Figure 1E).

Zebrafish as a host model enables studying interactions between patient cells and zebrafish innate immune cells (macrophages, neutrophils), endothelial cells, and erythrocytes/thrombocytes. Their transparency and transgenic reporters allow whole-body, single-cell resolution imaging, unlike mouse patient-derived xenografts. We observed that OC tumors were able to recruit zebrafish macrophages (Figure 1G), endothelial cells, and erythrocytes/thrombocytes into the TME (Figure 1H). This phenotype was transversal across OC tumor samples, from the PIs or from malignant effusions.

Overall, these results show that tumors in zAvatars preserved key features of the initial tumor sample, maintaining not only tumor cells but also some cells from the TME and recruitment of host immune and endothelial cells, creating a highly dynamic environment.[19]

Metastatic potential in zAvatars correlates with the initial patient prognosis

A major advantage of the zebrafish Avatar model is the possibility to study metastatic potential of tumor cells. In this assay,

(F–H) (F) Immunofluorescence characterization of zAvatar tumors at 3 dpi showing the presence of multiple cell types. Representative confocal images of (G) zebrafish macrophages (green) using *Tg(mpeg1.1:LOXP-DsRedx-LOXP-GFP)* and (H) zebrafish endothelial cells (green) and erythrocytes/thrombocytes (white) using *Tg(fli:EGFP)* and *Tg(fli:EGFP, gata1:RFP)* as hosts. Scale bars represent 10 μm. All immunofluorescence images are composites. MPE, malignant pleural effusions; OC, ovarian cancer; CAFs, cancer-associated fibroblasts; P, patient; hMITO, human mitochondria; ZF, zebrafish; AVG, average. See also Figure S1.

metastatic potential refers to the capacity of cancer cells to enter circulation, survive the shear stress of blood circulation, evade innate immune surveillance, and seed in different sites in the embryo.[20] Thus, the differential metastatic capacity observed across samples may be attributed to patient-specific tumor cell phenotypes and functional traits.

OC patients with high volume of malignant effusions are correlated with a poor prognosis.[21–23] We interrogated whether the zAvatars would reflect these differences in metastatic capacity and whether this could be linked to initial patient prognosis.

Within our cohort we had patients with only PIs and patients who had PIs concomitant with ascites (A) (PI-A) (Figure 2A). To evaluate the metastatic potential of tumor cells (in untreated zAvatars) derived from these 2 groups of patients, we generated zAvatars with 2 types of samples: (1) tissue from peritoneal implants, including omentum implants, of patients without malignant effusions (PI), and (2) tissue from the peritoneal implants of patients with concomitant ascites (PI-A). We scored several parameters at 3 dpi: (1) the incidence of zAvatars with micrometastases per patient, (2) the ability to disseminate to multiple metastatic sites, and (3) the pattern/tropism of the metastatic spread (Figures 2B–2E). OC patient cells can be detected at distant locations from the injection site, such as the head, eye, gills, or caudal hematopoietic (CHT) region (Figure 2B).

Our results show that tumor cells from the peritoneal implants of patients with malignant ascites (PI-A) exhibited significantly higher metastatic potential compared to those from patients without malignant ascites (PI) (**p = 0.008) (Figure 2C). Analysis of multiple metastatic sites revealed a significantly higher number of zAvatars with multiple metastatic spots in cells derived from patients with ascites, namely, to the head and CHT regions (Figures 2D and 2E).

Next, we questioned whether the tumor cells present in the ascites samples were representative of the PIs. To address this, we compared the behavior of cancer cells derived from the same patient but from different collection sites (PI-A vs. A). Our results show that in zAvatars, tumor cells behaved similarly in terms of tumor implantation and metastatic profile (Figures 2F and 2G). These results not only show that ascites were representative of the PIs but also reveals that cells derived from the peritoneal lining have similar metastatic capacities as the cells present in the ascites.

Our findings demonstrate that tumor cells in patients with malignant ascites exhibit augmented metastatic potential, indicating increased aggressiveness. Moreover, our results show that tumor cells in the ascites are representative of PIs, with similar metastatic capacities, suggesting that metastatic potential could be acquired prior to detachment from the peritoneal lining.

Ovarian cancer zAvatars predict patient's response to therapy

The major goal of this study was to conduct a co-clinical study to determine the predictive value of the OC zAvatar model. For this, we treated OC zAvatars from 32 patients with stage I to IV, with the same chemotherapy agents as the donor-patient and compared the patient clinical outcome with its matching OC zAvatar (Figures 3A and 3B). Patients in adjuvant setting who present a progression-free interval (PFI) greater than 6 months were considered platinum sensitive (responder), whereas patients with a PFI lower than 6 months were considered platinum resistant (non-responder).[24] In relapse patients, the response to treatment was evaluated by radiological assessment of tumor size and reduction in CA125 levels, as described in de Witte et al.[7]

After 3 days, the treatment-induced cytotoxicity in tumor cells was evaluated by quantifying cell death by apoptosis (activated-caspase 3 expression) in untreated vs. treated conditions (Figures 3C and 3D). As an example, in patient #3, carboplatin + paclitaxel (C + P) treatment led to a significant increase in apoptosis compared to untreated controls, correlating with a positive patient clinical response (Figure 3C). In contrast, in patient #6, there was no induction of apoptosis with C + P treatment, and correspondingly this patient progressed under therapy (Figure 3D).

Next, we plotted the zAvatar average induction of apoptosis of the 32 patients (expressed as fold change). Our results show that zAvatars from responder patients exhibited a significantly higher induction of apoptosis upon treatment, compared to zAvatars from non-responder patients (Figure 3E, ***$p < 0.001$). In contrast, when we plotted the tumor size or implantation fold change of responder vs. non-responder patients, we could not detect statistically differences between the 2 groups (Figures S2A and S2B). Thus, induction of apoptosis is the major readout to correlate the zAvatar-test with patient clinical outcome in OC.

To evaluate the ability of the zAvatar-test to separate clinical sensitivity and resistance, a Receiver Operating Characteristic analysis was performed using the average apoptosis fold change. Area under the curve was of 0.905, and a cutoff value of 1.6 was identified to determine sensitivity to the treatment (Figure S3). Based on this, a prediction matrix was generated to display the number of zAvatar-tests that accurately matched the patient clinical outcomes (Figure 3F). From a total of 19 patients with a sensitive zAvatar-test, 18 matched the patient clinical outcome, resulting in a positive predictive value (PPV) of 94.7%. Among the 13 patients in whom the zAvatar-test predicted resistance to treatment, 11 were indeed resistant to treatment, which corresponds to a negative predictive value (NPV) of 84.6%. Altogether, this corresponds to an overall accuracy of 91%.

zAvatar testing correlates with survival outcomes

To determine whether the zAvatar-test reflects patient survival outcomes, we plotted the PFS and OS into Kaplan-Meier survival curves of patients with zAvatar-sensitive test vs. patients with a zAvatar-resistant test (Figure 4A). The results demonstrate that patients with a sensitive zAvatar-test have significantly longer PFS (****$p < 0.0001$) and OS (**$p < 0.005$) than patients with a resistant zAvatar-test (Figures 4B and 4C). More specifically, patients with a zAvatar-sensitive test had median PFS of 17 months vs. 6 months in patients with a resistant test, whereas the median OS was 30 months in zAvatar-sensitive vs. 26 months in zAvatar-resistant test (Figures 4B and 4C). In other words, the PFS of patients with a sensitive test was 3 times longer than that of patients with a resistant test, whereas the OS was only 1.3 months longer.

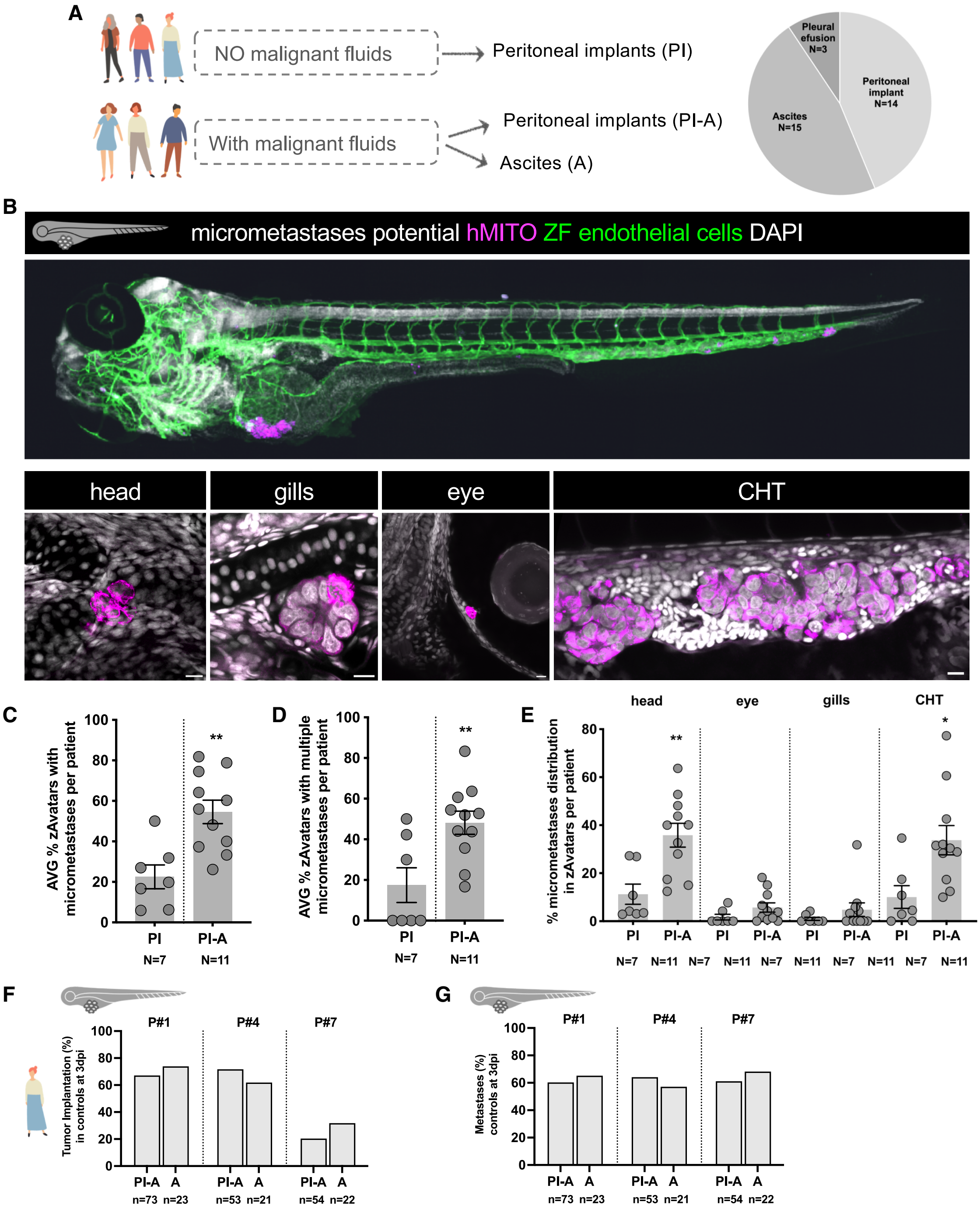

Figure 2. Metastatic potential of OC cells in zAvatars correlates with the patient prognosis

(A) Schematic illustration of tumor sample types used for the differential characterization of the metastatic burden.

(B–G) (B) Example of a zAvatar with micrometastases, where the final microscopy image was stitched together from individual panels. Representative confocal images of micrometastases detected in the head, gills, eye, and in CHT region of zAvatars at 3dpi. Analysis of (C) micrometastases incidence, (D) presence of micrometastases in multiple sites, and (E) distribution of tumor cells in the several anatomical locations in zAvatars generated from PI and PI-A. Results are expressed as AVG ± SEM. Each dot represents one zAvatar. Analysis of (F) tumor implantation and (G) micrometastases capacity of zAvatars generated from peritoneal implants of patients without ascites (PI) and with ascites (PI-A). Results are expressed as AVG. All datasets were analyzed using unpaired two-sided Mann-Whitney test (*$p < 0.01$;**$p < 0.001$; ns > 0.05). Scale bars represent 10 μm. All immunofluorescence images are composites. PI, peritoneal implants; A, ascites; PI-A, peritoneal implants with ascites; P, patient; hMITO, human mitochondria; AVG, average.

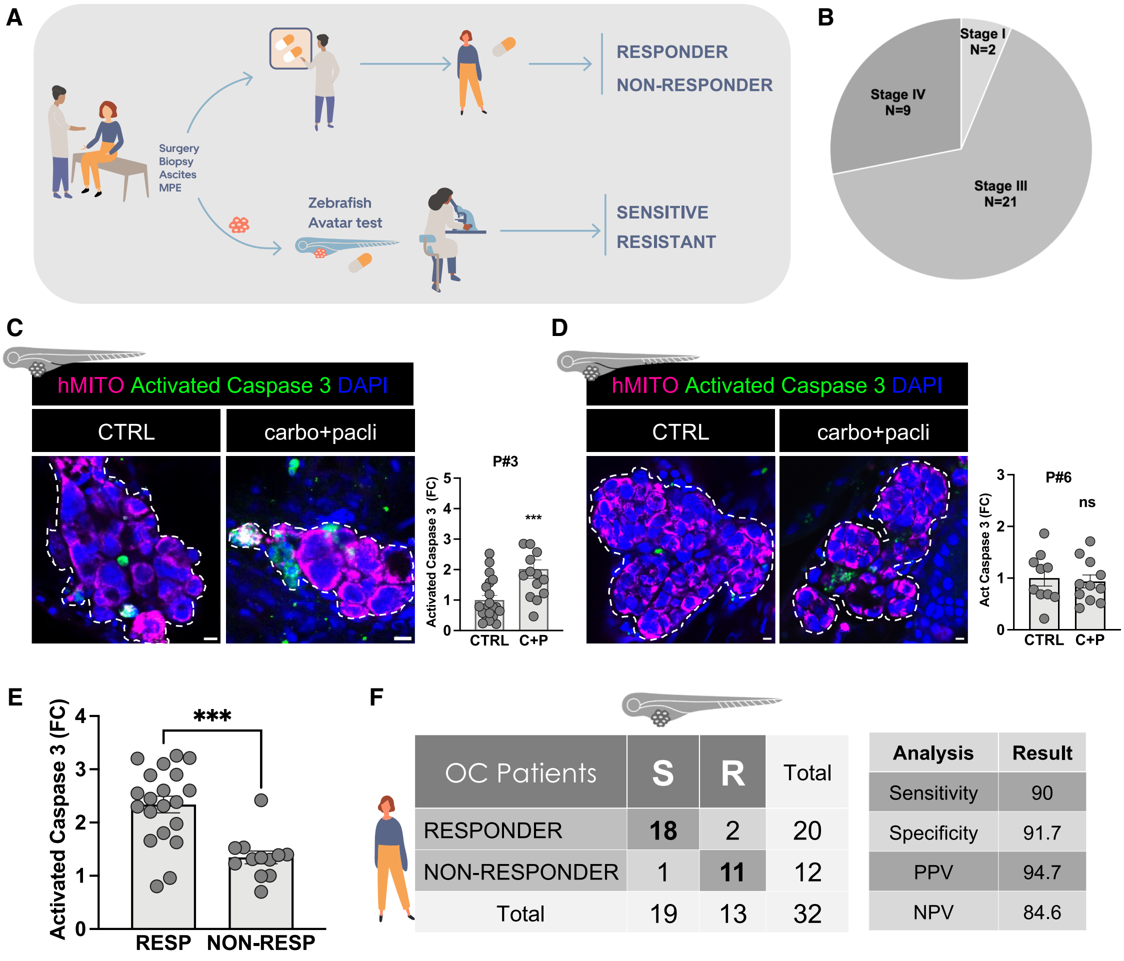

Figure 3. OC zAvatars predict patient response to chemotherapy

(A) Schematic illustration of the workflow used to evaluate the predictive power of zAvatars.

(B) Stage of OC patients included in this study.

(C and D) Confocal representative images of zAvatars tumors at 3 dpi showing tumor cells labeled with human mitochondria (magenta) and tumor cells expressing activated caspase-3 (green). Fold change of percentage of activated caspase-3 expression (***$p < 0.0001$; ns > 0.05). Each dot represents one zAvatar. All data are expressed as AVG ± SEM. Dashed white line delimits the tumor of each zAvatar. Scale bars represent 10 μm. All immunofluorescence images are composites.

(E) Fold change of percentage of activated caspase-3 expression of responders (RESP) and non-responders (NON-RESP). Each dot represents one patient. All data are expressed as AVG ± SEM. All datasets were analyzed using unpaired two-sided Mann-Whitney test (***$p < 0.0001$).

(F) Confusion matrix displays the number of patients with actual and predicted responses in zAvatars, i.e., sensitive (S) are zAvatars whose fold induction of apoptosis was >1.6, while zAvatars with fold induction of apoptosis ≤1.6 are classified as resistant (R). Carbo, carboplatin; Pacli, paclitaxel; CTRL, control; C + P, carboplatin plus paclitaxel; FC, fold change; RESP, responder; NON-RESP, non-responder. OC, ovarian cancer; S, sensitive; R, resistant; PPV, positive predictive value; NPV, negative predictive value. See also Figures S2 and S3.

BCL-2 inhibitors may overcome multidrug resistance in ovarian carcinomas

One of main advantages of using tumor samples from malignant effusions is the possibility to perform longitudinal profiling of tumor cells without additional interventions to the patient. We have shown that effusion-derived tumor cells mirrored the behavior of cells from solid lesions, validating their use for monitoring tumor dynamics. As such, we evaluated chemotherapy response and metastatic spread in 2 different time points (Figure 5A). This patient was diagnosed with stage IIIC high-grade serous OC and showed a multidrug resistant phenotype already in the adjuvant setting, which was maintained at the second relapse, since no cytotoxicity was observed to any of the therapies that were eligible according with the European Society for Medical Oncology guidelines (Figure 5B). In the control-zAvatars (untreated), the metastatic burden increased from 33% to 79% (first line vs. second relapse, respectively) and was not decreased by any of the treatments (Figure 5C). The ability to generate zAvatars from longitudinal sampling of OC patients confirms that, as the tumor progresses, cells become more metastatic and drug resistance is maintained or aggravated.

Multidrug resistance significantly impacts the prognosis of OC patients. This phenomenon can be triggered by several mechanisms, including the alteration of apoptotic pathways, which

CellPress
OPEN ACCESS

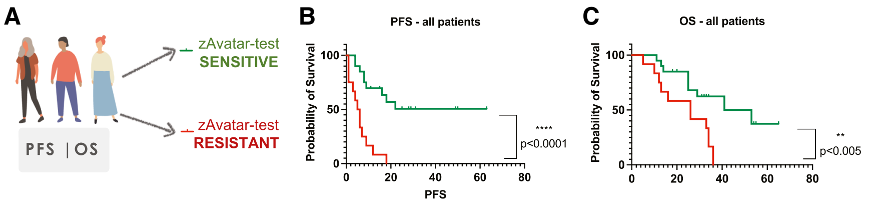

Figure 4. zAvatar testing correlates with survival outcomes

(A) Kaplan-Meier survival curves to compare the PFS and OS of patients based on sensitivity or resistance of their zAvatar-test. Kaplan-Meier for PFS (B) and OS (C). The PFS was calculated from the initiation of therapy until last observation or disease progression (N = 32 patients; ****$p < 0.0001$). The OS was calculated from the date of diagnosis until last observation or death (N = 32 patients; **$p < 0.005$). PFS, progression free survival; OS, overall survival. See also Figure S4.

allows tumor cells to evade chemotherapy-induced cell death.[25,26] One example is the overexpression of the anti-apoptotic BCL2 protein that has been associated with this phenotype.[26] As such we tested the BCL2 inhibitor Venetoclax (Vx), approved and in use for chronic lymphocytic leukemia and small lymphocytic lymphoma in a different multidrug-resistant tumor sample. zAvatars were treated with Vx alone or in combination with C + P (Figures 5D–5F). As expected, C + P had no cytotoxic effect. However, Vx alone significantly induced activated caspase-3 expression in the tumor (**$p = 0.0025$; Figure 5E) and this effect was even more pronounced in the combination of Vx with C + P, increasing activated caspase-3 to 1.85-fold (***$p = 0.0008$; Figure 5E). Although not statistically significant, the C + P + Vx combination therapy led to a reduction in the metastatic burden, decreasing the proportion of multiple metastatic sites from 42% in the control group to 26% in the C + P + Vx group (Figure 5F). To complement our analysis, and as an alternative visualization of the results, we performed a pixel wise sum of all slices (SUM projection) of the activated caspase-3 in the region of interest defined by the human-mitochondria-positive cells, in each zAvatar of the four conditions tested (Figure 5G). The results confirm that there is a higher signal intensity of activated caspase-3 in the C + P + Vx condition, compared with the control or with the C + P alone.

Altogether, these results suggest that multidrug-resistant OC might be sensitized by BCL-2 inhibitors, which could be considered in the future as an off-label option alone or in combination with chemotherapy for patients with resistant disease.

DISCUSSION

OC is the main cause of death among gynecological cancers. When patients relapse, there are equivalent treatment options available, leaving patients and oncologists with the weight of choosing which therapy to give, with limited biomarkers to guide treatment decisions. When the treatment is ineffective, not only patients are subjected to toxicities but also a favorable environment is created for tumor progression and the development of multidrug-resistant states.[8]

The promise of personalized medicine has led to an array of patient-derived models, aiming to predict patient response to therapy and thus, guide patient therapy. The gold-standard mouse patient-derived xenografts (mAvatars) have good predictive values (*N* = 13/16 patients, 81% predictive value).[27] However, generation and treatment of mAvatars can take months, which is not compatible with timings for clinical decisions.[28–30] Alternatively, *in vitro* 3D cancer organoids and spheroids are also emerging, although the predictive value of OC organoids was not yet determined.[7,31] Still, these models lack the complexity of a living organism, limiting the test of several therapies (antiangiogenic, or therapies that need *in vivo* metabolization) and, more important, do not allow the evaluation of the tumor metastatic potential.[30] Nonetheless, the use of zebrafish might also present some limitations. The primary limitation of the zAvatar-test is its reliance on fresh tumor tissue, which limits the number of therapies that can be evaluated from a single sample. This constraint arises because patient-derived zAvatars are generated without *in vitro* or *in vivo* amplification. In OC, however, this is less restrictive, since interval debulking surgeries often yield substantial tumor tissue and many patients present with high-volume malignant effusions, enabling the collection of sufficient tumor cells. Another limitation is the requirement to maintain zAvatars at 34°C, which may reduce proliferation rates. Nevertheless, our prior studies have demonstrated that human cells can proliferate in zebrafish at 34°C, with results translatable to humans, as evidenced by the high predictive accuracy of zAvatars in colorectal and breast cancers,[9,10,32] and now in this OC study.

The zebrafish patient-derived xenograft—zAvatar model—has been gaining attention, and 2 recent studies, with a small cohort of patients, have reported different predictive values (81%, with 13 patients[33] and 100% in 6 patients[34]).

In the present study, we achieved a predictive value of 91%, with a PPV of 95% and an NPV of 85%, with the largest cohort of patients (*N* = 32 patients), when compared to zAvatars, mAvatars, or organoids.[2,27,34] A major difference between our study and the previous OC zAvatars studies, besides the cohort size, is the methodology based on single-cell confocal analysis of apoptosis. Moreover, in this study, due to the cohort size we were able to compare PFS and OS; patients with an OC zAvatar-sensitive test had a significantly longer PFS and OS compared to patients with a resistant zAvatar-test.

To characterize the model, we started by showing that tumors in zAvatars are representative of the initial tumor sample and that tumor-stroma crosstalk was achieved with zebrafish host cells, namely, with macrophages and endothelial cells. More specifically, we observed that macrophages infiltrated and phagocytosed tumor cells and that endothelial cells are recruited and encapsulate tumor cells forming small clusters. One reason might be the high amounts of VEGF that OC cells secrete, which induce endothelial cell recruitment to the tumor.[35] Together, this demonstrates that tumors in zAvatars are highly complex and dynamic, which more accurately reflect the complex biology of tumors in patients. This complexity is critical for assessing

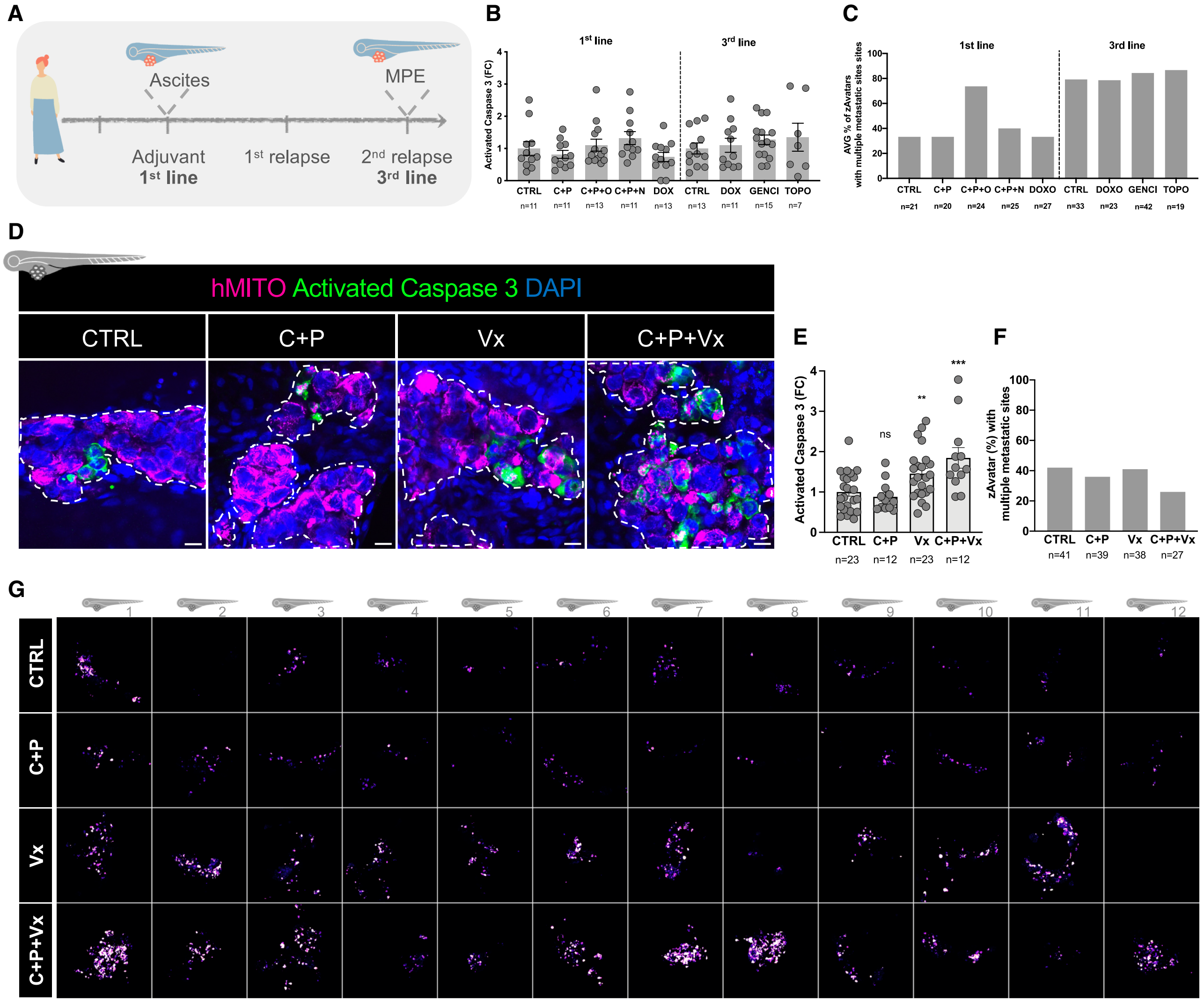

Figure 5. BCL-2 inhibitors may overcome multidrug resistance in ovarian carcinomas

(A) Schematic illustration of longitudinal sampling of an OC patient for generation of zAvatars.

(B) Fold change of percentage of activated caspase-3 expression in the adjuvant setting and in the second relapse. Data are expressed as AVG ± SEM.

(C) Analysis of zAvatars with metastases in multiple sites in zAvatars generated with tumor cells from the same patient in a more advanced stage.

(D) Confocal representative images of zAvatar tumors at 3 dpi, showing tumor cells labeled with human mitochondria (magenta) and tumor cells expressing activated caspase-3 (green). Dashed white line delimitates the tumor of each zAvatar. Scale bars represent 10 μm. All immunofluorescence images are composites.

(E) Fold change of percentage of activated caspase-3 expression (** p = 0.0025, *** p = 0.0008). Data are expressed as AVG ± SEM. Data were analyzed using unpaired two-sided Mann-Whitney test. Each dot represents one zAvatar.

(F) Analysis of the percentage of zAvatars with metastases in multiple sites. Data are expressed as AVG.

(G) Representative images of SUM projections of the activated caspase-3 in the region of interest defined by the human-mitochondria positive cells in each zAvatar of the four conditions tested. Each square represents a different zAvatar. FC, fold change; CTRL, control; C + P, carboplatin plus paclitaxel. C + P + O, carboplatin plus paclitaxel plus olaparib; C + P + N, carboplatin + paclitaxel + niraparib; DOX, doxorubicin, GENCI, gemcitabine; TOPO, topotecan; Carbo, carboplatin; Pacli, paclitaxel; Vx, venetoclax; C + P + Vx, carboplatin plus paclitaxel plus venetoclax; AVG, average.

drug responses because tumors *in vivo* interact with diverse cell types and signaling pathways, influencing drug efficacy and resistance.[19] Besides the high predictive value of the OC zAvatar model, we showed that the metastatic burden in zAvatars can be correlated with the patient's initial prognosis. Specifically, cells from PIs in patients with malignant effusion showed higher metastatic potential compared to cells from patients without malignant effusion. Moreover, the metastatic potential was similar between cells from PIs and those from ascites. Although patients with a high volume of ascites have worse overall survival,[21–23] they receive the same follow-up. Overall, our findings suggest that tumor cells in PIs may contribute to malignant effusion development, showing a more aggressive phenotype than cells from patients without malignant effusions. zAvatars' sensitivity

for assessing tumor cell aggressiveness can be a *red flag* to patients with a poorer prognosis, who need a closer follow-up.

Multidrug-resistant phenotype is a major concern in the management of OC patients. The treatment of these patients is a major challenge and raises the question if it is possible to transform "non-responder" patients into "responders," which could significantly change the course of the disease. To test this hypothesis, we treated a multidrug-resistant tumor with the BCL2 inhibitor Vx, as BCL-2-family anti-apoptotic proteins have been associated with chemo-resistant phenotypes in OC.[26] Vx led to a significant increase in apoptosis, especially when combined with C + P, showing that by inhibiting the anti-apoptotic protein BCL-2 it is possible to sensitize multidrug-resistant tumor cells to chemotherapy. Altogether, these results suggest that the OC zAvatar-test can be used to screen off-label treatments for multidrug-resistant patients.

To make personalized medicine a reality and bring the predictive power of the zAvatar-test to patients, a clinical trial is mandatory. As such, based on these findings we have started a clinical trial to test the clinical benefit of using zAvatars by evidence-based medicine. We expect that, by the end of this trial, we will be able to demonstrate that the use of the zAvatar-test can prolong the PFS and OS of OC patients. We believe that the implementation of a functional precision oncology test in the clinic, such as the zAvatar-test, will transform patient care, optimizing all treatments, increasing the life expectancy, and reducing the burden on healthcare systems. Although the existing manual pipeline is sufficient for proof of concept studies, it lacks the scalability and the efficiency required for integration into clinical practice. As a next step, we are developing an AI-based high-throughput analysis system that will not only reduce the time of analysis but also increase the reproducibility and throughput of the assay. This will allow us to scale-up in a robust manner and take the zAvatar test to more patients worldwide.

Limitations of the study

While our study shows promising results for the use of zAvatars as a companion diagnostic test for OC patients, there are aspects that still need further addressing. First, our cohort of patients is mostly composed by patients undergoing first-line of therapy (25/32 patients) and only a small cohort of 7/32 patients were in relapse. Since patients in relapse are our major target population, because it is where the equivalent treatment options arise, it would be important to increase these numbers in future studies. Second, the predictive value of the zAvatar-test was determined with carboplatin-based regimens and only one patient in the cohort was treated with paclitaxel alone. Finally, the clinical benefit of the zAvatar-test still remains to be determined, which is why we are conducting a multicentric randomized clinical trial with advanced OC and metastatic breast cancer patients.

RESOURCE AVAILABILITY

Lead contact

Requests for further information and resources should be directed to and will be fulfilled by the lead contact, Rita Fior, PhD, (rita.fior@research.fchampalimaud.org).

Materials availability

This study did not generate new unique reagents.

Data and code availability

- Data: The authors declare that the main data supporting the findings of this study are available within the article and in the supplemental information (Table S1; Table S7). Raw immunofluorescence confocal images generated in this study are available from the lead contact with a completed materials transfer agreement. Other data related to this work, including datasets on the fold change of apoptosis, tumor size, and metastatic potential from all zAvatars analyzed across the 32 patients, are available from the lead contact upon request.
- Code: This paper does not report original code.
- General statement: Any additional information required to reanalyze the data reported in this work paper is available from the lead contact upon request.

ACKNOWLEDGMENTS

We acknowledge all patients who supported our research by consenting access to clinical specimens. We also acknowledge the clinical and administrative team of the Gynecology Unit and nurse Vanda Câmara, from the Radiology Unit of the Champalimaud Foundation, who always dedicated time to help and contribute to this research. This work was funded by the Champalimaud Foundation, by the Portuguese Foundation for Science and Technology (PTDC/BTM-SAL/3796/2021), by Congento (LISBOA-01-0145-FEDER-022170, co-financed by FCT/Lisboa2020), by the BIAL Award in Clinical Medicine, and by the funding Swiss Cancer Research (#KFS-5389-08-2021-R to F.J.). We thank the Champalimaud Fish Platform, the Surgery Departments and Pathology Services at the Champalimaud Clinical Center. We acknowledge Marta Correia for the illustration of the graphical abstract. We are also grateful to Martim Gamboa, who contributed to Figure 5. We also acknowledge Dr. Saudade André and Dr. Ruben Roque, from Instituto Português de Oncologia de Lisboa Francisco Gentil, for contributing with the PEG protocols to perform immunofluorescence analysis in cell smears. We dedicate this work to the memory of Dr. Henrique Nabais, whose vision, knowledge, and commitment were a driving force behind this study and a constant motivation to the team.

AUTHOR CONTRIBUTIONS

M.F.E. conceptualized, performed, and analyzed all research and acquired funding. R.F. conceptualized, supervised the research, and acquired funding. F.A., C.R.d.A., and M.F. performed research. F.F.d.S., M.d.L.B., and H.N. participated in selecting and analyzing patient data. R.C., F.J., V.H.-S., J.C., A.L., E.Y.-G., A.L., R.C.M., M.C.-M., and H.N. provided patient tumor samples. S.F. collected signed informed consents from patients. A.M. supervised statistical analysis. M.F.E. and R.F. wrote the manuscript. All authors contributed with the critical reading of the manuscript. All authors have read and agreed to the published version of the manuscript.

DECLARATION OF INTERESTS

The authors declare no competing interests.

STAR★METHODS

Detailed methods are provided in the online version of this paper and include the following:

- KEY RESOURCES TABLE
- EXPERIMENTAL MODEL AND STUDY PARTICIPANT DETAILS
  - Patient clinical data and follow up
  - Zebrafish model
- METHOD DETAILS
  - Patient sample processing for cryopreservation
  - Patient sample processing for zebrafish microinjection

- ○ Zebrafish patient-derived xenograft microinjection
- ○ Drug administration
- ○ Whole-mount immunofluorescence
- ○ Immunofluorescence in smears
- ○ Imaging and quantification
- ● QUANTIFICATION AND STATISTICAL ANALYSIS

SUPPLEMENTAL INFORMATION

Supplemental information can be found online at https://doi.org/10.1016/j.xcrm.2025.102530.

Received: August 1, 2025
Revised: October 6, 2025
Accepted: November 21, 2025
Published: December 30, 2025

REFERENCES

1. Ghoneum, A., Afify, H., Salih, Z., Kelly, M., and Said, N. (2018). Role of tumor microenvironment in ovarian cancer pathobiology. Oncotarget *9*, 22832–22849. https://doi.org/10.18632/oncotarget.25126.
2. Kopper, O., de Witte, C.J., Lõhmussaar, K., Valle-Inclan, J.E., Hami, N., Kester, L., Balgobind, A.V., Korving, J., Proost, N., Begthel, H., et al. (2019). An organoid platform for ovarian cancer captures intra- and inter-patient heterogeneity. Nat. Med. *25*, 838–849. https://doi.org/10.1038/s41591-019-0422-6.
3. Zhang, Y., Cao, L., Nguyen, D., and Lu, H. (2016). TP53 mutations in epithelial ovarian cancer. Transl. Cancer Res. *5*, 650–663. https://doi.org/10.21037/tcr.2016.08.40.
4. Ford, C.E., Werner, B., Hacker, N.F., and Warton, K. (2020). The untapped potential of ascites in ovarian cancer research and treatment. Br. J. Cancer *123*, 9–16. https://doi.org/10.1038/s41416-020-0875-x.
5. Redondo, A., Guerra, E., Manso, L., Martin-Lorente, C., Martinez-Garcia, J., Perez-Fidalgo, J.A., Varela, M.Q., Rubio, M.J., Barretina-Ginesta, M.P., and González-Martín, A. (2021). SEOM clinical guideline in ovarian cancer (2020). Clin. Transl. Oncol. *23*, 961–968. https://doi.org/10.1007/s12094-020-02545-x.
6. Pignata, S., Pisano, C., Di Napoli, M., Cecere, S.C., Tambaro, R., and Attademo, L. (2019). Treatment of recurrent epithelial ovarian cancer. Cancer *125*, 4609–4615. https://doi.org/10.1002/cncr.32500.
7. de Witte, C.J., Espejo Valle-Inclan, J., Hami, N., Lõhmussaar, K., Kopper, O., Vreuls, C.P.H., Jonges, G.N., van Diest, P., Nguyen, L., Clevers, H., et al. (2020). Patient-Derived Ovarian Cancer Organoids Mimic Clinical Response and Exhibit Heterogeneous Inter- and Intrapatient Drug Responses. Cell Rep. *31*, 107762. https://doi.org/10.1016/j.celrep.2020.107762.
8. Ibragimova, M.K., Tsyganov, M.M., and Litviakov, N.V. (2017). Natural and Chemotherapy-Induced Clonal Evolution of Tumors. Biochemistry. *82*, 413–425. https://doi.org/10.1134/S0006297917040022.
9. Fior, R., Póvoa, V., Mendes, R.V., Carvalho, T., Gomes, A., Figueiredo, N., and Ferreira, M.G. (2017). Single-cell functional and chemosensitive profiling of combinatorial colorectal therapy in zebrafish xenografts. Proc. Natl. Acad. Sci. USA *34*, 201618389. https://doi.org/10.1073/pnas.1618389114.
10. Costa, B., Estrada, M.F., Gomes, A., Fernandez, L.M., Azevedo, J.M., Póvoa, V., Fontes, M., Alves, A., Galzerano, A., Castillo Martin, M., et al. (2024). Zebrafish Avatar-test forecasts clinical response to chemotherapy in patients with colorectal cancer. Nat. Commun. *15*, 4771. https://doi.org/10.1038/s41467-024-49051-0.
11. Póvoa, V., Rebelo de Almeida, C., Maia-Gil, M., Sobral, D., Domingues, M., Martinez-Lopez, M., de Almeida Fuzeta, M., Silva, C., Grosso, A.R., and Fior, R. (2021). Innate immune evasion revealed in a colorectal zebrafish xenograft model. Nat. Commun. *12*, 1156. https://doi.org/10.1038/s41467-021-21421-y.
12. Martínez-López, M.F., de Almeida, C.R., Fontes, M., Mendes, R.V., Kaufmann, S.H.E., and Fior, R. (2024). Macrophages directly kill bladder cancer cells through TNF signaling as an early response to BCG therapy. Dis. Model. Mech. *17*, dmm050693. https://doi.org/10.1242/dmm.050693.
13. Soriano, A.A., de Cristofaro, T., Di Palma, T., Dotolo, S., Gokulnath, P., Izzo, A., Calì, G., Facchiano, A., and Zannini, M. (2019). PAX8 expression in high-grade serous ovarian cancer positively regulates attachment to ECM via Integrin β3. Cancer Cell Int. *19*, 303–312. https://doi.org/10.1186/s12935-019-1022-8.
14. Liu, J., Li, L., Luo, N., Liu, Q., Liu, L., Chen, D., Cheng, Z., and Xi, X. (2021). Inflammatory signals induce MUC16 expression in ovarian cancer cells via NF-κB activation. Exp. Ther. Med. *21*, 163. https://doi.org/10.3892/etm.2020.9594.
15. Wang, J., Zohar, R., and McCulloch, C.A. (2006). Multiple roles of alpha-smooth muscle actin in mechanotransduction. Exp. Cell Res. *312*, 205–214. https://doi.org/10.1016/j.yexcr.2005.11.004.
16. Chistiakov, D.A., Killingsworth, M.C., Myasoedova, V.A., Orekhov, A.N., and Bobryshev, Y.V. (2017). CD68/macrosialin: not just a histochemical marker. Lab. Invest. *97*, 4–13. https://doi.org/10.1038/labinvest.2016.116.
17. Spour, E.F., Leemhuis, T., Jenski, L., Redmond, R., Fillak, D., and Jansen, J. (1990). Characterization of normal human CD3+ CD5- and gamma delta T cell receptor positive T lymphocytes. Clin. Exp. Immunol. *80*, 114–121. https://doi.org/10.1111/j.1365-2249.1990.tb06450.x.
18. Veneziani, A.C., Gonzalez-Ochoa, E., Alqaisi, H., Madariaga, A., Bhat, G., Rouzbahman, M., Sneha, S., and Oza, A.M. (2023). Heterogeneity and treatment landscape of ovarian carcinoma. Nat. Rev. Clin. Oncol. *20*, 820–842. https://doi.org/10.1038/s41571-023-00819-1.
19. Bissell, M.J., and Hines, W.C. (2011). Why don't we get more cancer? A proposed role of the microenvironment in restraining cancer progression. Nat. Med. *17*, 320–329. https://doi.org/10.1038/nm.2328.
20. Rebelo de Almeida, C., Mendes, R.V., Pezzarossa, A., Gago, J., Carvalho, C., Alves, A., Nunes, V., Brito, M.J., Cardoso, M.J., Ribeiro, J., et al. (2020). Zebrafish xenografts as a fast screening platform for bevacizumab cancer therapy. Commun. Biol. *3*, 299. https://doi.org/10.1038/s42003-020-1015-0.
21. Nasioudis, D., Byrne, M., Ko, E.M., Haggerty, A.F., Cory, L., Giuntoli Ii, R.L., Kim, S.H., and Latif, N.A. (2021). Ascites volume at the time of primary debulking and overall survival of patients with advanced epithelial ovarian cancer. Int. J. Gynecol. Cancer *31*, 1579–1583. https://doi.org/10.1136/ijgc-2021-002978.
22. Quan, Q., Zhou, S., Liu, Y., Yin, W., Liao, Q., Ren, S., Zhang, F., Meng, Y., and Mu, X. (2021). Relationship between ascites volume and clinical outcomes in epithelial ovarian cancer. J. Obstet. Gynaecol. Res. *47*, 1527–1535. https://doi.org/10.1111/jog.14682.
23. Yoshihara, M., Emoto, R., Kitami, K., Iyoshi, S., Uno, K., Mogi, K., Tano, S., Yoshikawa, N., Matsui, S., and Kajiyama, H. (2021). A large-scale multi-institutional study evaluating prognostic aspects of positive ascites cytology and effects of therapeutic interventions in epithelial ovarian cancer. Sci. Rep. *11*, 15154–15158. https://doi.org/10.1038/s41598-021-93718-3.
24. Pfisterer, J., and Ledermann, J.A. (2006). Management of platinum-sensitive recurrent ovarian cancer. Semin. Oncol. *33*, S12–S16. https://doi.org/10.1053/j.seminoncol.2006.03.012.
25. Williams, J., Lucas, P.C., Griffith, K.A., Choi, M., Fogoros, S., Hu, Y.Y., and Liu, J.R. (2005). Expression of Bcl-xL in ovarian carcinoma is associated with chemoresistance and recurrent disease. Gynecol. Oncol. *96*, 287–295. https://doi.org/10.1016/j.ygyno.2004.10.026.
26. Yokoyama, T., Kohn, E.C., Brill, E., and Lee, J.-M. (2017). Apoptosis is augmented in high-grade serous ovarian cancer by the combined inhibition of Bcl-2/Bcl-xL and PARP. Int. J. Oncol. *50*, 1064–1074. https://doi.org/10.3892/ijo.2017.3914.

CellPress
OPEN ACCESS

27. Izumchenko, E., Paz, K., Ciznadija, D., Sloma, I., Katz, A., Vasquez-Dunddel, D., Ben-Zvi, I., Stebbing, J., McGuire, W., Harris, W., et al. (2017). Patient-derived xenografts effectively capture responses to oncology therapy in a heterogeneous cohort of patients with solid tumors. Ann. Oncol. *28*, 2595–2605. https://doi.org/10.1093/annonc/mdx416.

28. Costa, B., Estrada, M.F., Mendes, R.V., and Fior, R. (2020). Zebrafish Avatars towards Personalized Medicine-A Comparative Review between Avatar Models. Cells *9*, 293. https://doi.org/10.3390/cells9020293.

29. Bose, S., Barroso, M., Chheda, M.G., Clevers, H., Elez, E., Kaochar, S., Kopetz, S.E., Li, X.-N., Meric-Bernstam, F., Meyer, C.A., et al. (2022). A path to translation: How 3D patient tumor avatars enable next generation precision oncology. Cancer Cell *40*, 1448–1453. https://doi.org/10.1016/j.ccell.2022.09.017.

30. Bose, S., Clevers, H., and Shen, X. (2021). Promises and Challenges of Organoid-Guided Precision Medicine. Med *2*, 1011–1026. https://doi.org/10.1016/j.medj.2021.08.005.

31. Nero, C., Vizzielli, G., Lorusso, D., Cesari, E., Daniele, G., Loverro, M., Scambia, G., and Sette, C. (2021). Patient-derived organoids and high grade serous ovarian cancer: from disease modeling to personalized medicine. J. Exp. Clin. Cancer Res. *40*, 116. https://doi.org/10.1186/s13046-021-01917-7.

32. Mendes, R.V., Ribeiro, J.M., Gouveia, H., Rebelo de Almeida, C., Castillo Martin, M., Brito, M.J., Canas-Marques, R., Batista, E., Alves, C., Sousa, B., et al. (2025). Zebrafish Avatar testing preclinical study predicts chemotherapy response in breast cancer. npj Precis. Oncol. *9*, 94. https://doi.org/10.1038/s41698-025-00882-0.

33. Song, F., Yi, X., Zheng, X., Zhang, Z., Zhao, L., Shen, Y., Zhi, Y., Liu, T., Liu, X., Xu, T., et al. (2025). Zebrafish patient-derived xenograft system for predicting carboplatin resistance and metastasis of ovarian cancer. Drug Resist. Updat. *78*, 101162. https://doi.org/10.1016/j.drup.2024.101162.

34. Lindahl, G., Fjellander, S., Selvaraj, K., Vildeval, M., Ali, Z., Almter, R., Erkstam, A., Rodriguez, G.V., Abrahamsson, A., Kersley, Å.R., et al. (2024). Zebrafish tumour xenograft models: a prognostic approach to epithelial ovarian cancer. npj Precis. Oncol. *8*, 53. https://doi.org/10.1038/s41698-024-00550-9.

35. Rickard, B.P., Conrad, C., Sorrin, A.J., Ruhi, M.K., Reader, J.C., Huang, S.A., Franco, W., Scarcelli, G., Polacheck, W.J., Roque, D.M., et al. (2021). Malignant Ascites in Ovarian Cancer: Cellular, Acellular, and Biophysical Determinants of Molecular Characteristics and Therapy Response. Cancers (Basel) *13*, 4318. https://doi.org/10.3390/cancers13174318.

36. Lawson, N.D., and Weinstein, B.M. (2002). In vivo imaging of embryonic vascular development using transgenic zebrafish. Dev. Biol. *248*, 307–318. https://doi.org/10.1006/dbio.2002.0711.

37. He, S., Chen, J., Jiang, Y., Wu, Y., Zhu, L., Jin, W., Zhao, C., Yu, T., Wang, T., Wu, S., et al. (2018). Adult zebrafish Langerhans cells arise from hematopoietic stem/progenitor cells. eLife *7*, e36131. https://doi.org/10.7554/eLife.36131.

38. Deng, S., Wu, Q., Zhao, Y., Zheng, X., Wu, N., Pang, J., Li, X., Bi, C., Liu, X., Yang, L., et al. (2015). Biodegradable polymeric micelle-encapsulated doxorubicin suppresses tumor metastasis by killing circulating tumor cells. Nanoscale *7*, 5270–5280. https://doi.org/10.1039/c4nr07641a.

STAR★METHODS

KEY RESOURCES TABLE

REAGENT or RESOURCE	SOURCE	IDENTIFIER
**Antibodies**		
Anti-Human mitochondria, clone 113- 1	Merck Millipore	Cat# MAB1273; RRID:AB_94052
Anti-Caspase 3, clone 5A1E	Cell Signaling	Cat# 9661; RRID:AB_2341188
Anti-PAX8, clone EPR18715	Abcam	Cat# ab191870
Anti-CA125, clone OV185:1	Leica Biosystems	Cat# CA125-L-CE
Anti-CD68, clone 514H12	Leica Biosystems	Cat# CD68-L-CE
Anti-aSMA, clone EPR5368	Abcam	Cat# ab124964: RRID:AB_11129103
Anti-CD3, clone LN10	Leica Biosystems	Cat# CD3-565-L-CE
Anti-CD5, clone 4C7	Leica Biosystems	Cat# CD5-4C7-L-CE
Anti-mouse 488 DyLight™	ThermoFisher Scientific	Cat# 35502
Anti-mouse 594 DyLight™	ThermoFisher Scientific	Cat# 35510
Anti-rabbit 488 DyLight™	ThermoFisher Scientific	Cat# 35552
Anti-rabbit 594 DyLight™	ThermoFisher Scientific	Cat# 35560
**Biological samples**		
Patient-derived ovarian cancer samples from surgical resection or malignant effusions	Champalimaud Foundation clinical center	N/A
**Chemicals, peptides, and recombinant proteins**		
Dimethyl sulfoxide (DMSO)*	ThermoFisher Scientific	10103483
Fetal Bovine Serum (FBS)*	Sigma	F7524
Red-blood cell lysis buffer (RBCLB)	Abcam	ab204733
Advanced DMEM/F12	ThermoFisher Scientific	12634010
Lypophilic dye – CellTracker Deep Red Dye	ThermoFisher Scientific	C34565
100 U/mL Penicillin-Streptomycin	ThermoFisher Scientific	15140122
Pierce™ 16% Formaldehyde (w/v), Methanol-free	ThermoFisher Scientific	28906
Metanol ≥99.8% AnalaR NORMAPUR® ACS	VWR	20847.320
Agar for bacteriology	VWR	97064–336
1% (v/v) HEPES	Corning	MT25015CI
MEM Non-Essential Amino Acids Solution (NEA	ThermoFisher Scientific	11140050
1x B-27™ Supplement	ThermoFisher Scientific	17504044
10 μg/mL Putrescine dihydrochloride	Sigma-Aldrich	P5780
10 mM Nicotinamide	Sigma-Aldrich	72340
1.25 mM N-acetylcysteine	Sigma-Aldrich	A9165
0.5 μg/mL Hydrocortisone*	Sigma-Aldrich	H0888
glutaGRO Supplement	Corning	MT25015CI
1% (v/v) Insulin-Transferrin-Selenium (ITS)*	Corning	15383661
10 μM Y-27632	Selleckchem	S6390
50 ng/mL EGF	Peprotech	AF-100-15
100 μg/mL Primocin	Invivogen	ant-pm-1
37.5 U/mL Universal Nuclease	Pierce	88700
Tricaine powder	Sigma-Aldrich	886-86-2
DAPI	Sigma-Aldrich	10236276001

(*Continued on next page*)

Continued

REAGENT or RESOURCE	SOURCE	IDENTIFIER
Goat Serum	Sigma-Aldrich	G9023
Mowiol	Sigma-Aldrich	81381
Poly(ethylene glycol)*	Sigma-Aldrich	P5402
Triton X-100	Thermofisher Scientific	10254583
Entellan	Merck Millipore	1079600500
Bovine serum albumin Fraction V	Carl Roth	T844.2
Tween 20	Fisher Scientific	BP337-100
Critical commercial assays		
Giemsa Reagents	Bio-Optica	04-090805M
Experimental models: Organisms/strains		
Zebrafish (*Danio rerio*) *Tg(fli1:EGFP)*	Lawson et al.[36]	ZDB-ALT-011017-8
Zebrafish (*Danio rerio*) *Tg(mpeg1.1: LOXP-DsRedx-LOXP-GFP)*	Sicong He et al.[37]	ZDB-TGCONSTRCT-181207-7
Zebrafish (*Danio rerio*) Tg(*fli1:EGFP*, *gata1:RFP)*	Lawson et al.[36]; Deng et al.[38]	Outcross between 2 transgenic lines<br>Gata: ZDB-TGCONSTRCT-161214-4<br>Fli: ZDB-ALT-011017-8
Software and algorithms		
GraphpPad Prism	GraphpPad	https://www.graphpad.com
ZEN	Carl Zeiss	https://www.zeiss.com/microscopy/int/products/microscope-software/zen.html
SPSS	IBM SPSS software	https://www.ibm.com/products/spss

EXPERIMENTAL MODEL AND STUDY PARTICIPANT DETAILS

Patient clinical data and follow up

The study was approved by the Ethics Committee of the Champalimaud Foundation, by the Ethical Committee of the Ethikkommission Nordwest-und Zentralschweiz (EKNZ; BASEC ID 2023-00988, VHS). All patients have signed an Informed Consent Form (ICF) to agree to donate the material for research purposes. All samples were anonymized. The inclusion criteria were as follows: women aged ≥18 years, who were able to understand the study procedures and to provide a signed written ICF, for whom there is a decision for therapy treatment. Patients were treated according to the standard of care based on European Society for Medical Oncology (ESMO) clinical guidelines decided in a multidisciplinary team (MDT) meeting. Clinical data included surgical and pathological data and BRCA status, (Table S1). The follow-up data included the type of chemotherapy, number of cycles and duration, metastases incidence, presence of malignant effusions (ascites or pleural effusion), PFS (calculated from the date of treatment initiation until the date of disease progression or death) and OS (calculated from the date of diagnosis until last follow up or death). Follow-up of ovarian cancer patients was performed according to ESMO guidelines, with clinical evaluation every 3 to 6 months. Classification of progression/no-progression disease was based in CA125 levels, imagological assessment computed tomography (CT), magnetic resonance imaging (MRI) and/or positron emission tomography (PET), clinical assessment, all discussed in MDT meetings. Patients in the adjuvant setting (adjuvant or neoadjuvant chemotherapy) were classified as “responders” if relapsed occurred in more than 6 months post chemotherapy (platinum-sensitive), whereas patients that relapsed in less than 6 months post chemotherapy, were considered “non-responders” (platinum-resistant patients) [13]. For patients in this study who were already experiencing a relapse and were not eligible for surgical resection, the response to therapy was assessed through radiological evaluation of tumor size and/or by measuring reductions in CA125 levels [7]. Response to zAvatar treatment was compared with the patient’s clinical response. Experimental researchers had no previous information about the clinical outcome. After performing the zAvatar-test, results were sent to the physicians for correlation analysis.

Zebrafish model

In vivo experiments were performed using the zebrafish (Danio rerio) model, which was handled according to European animal welfare Legislation, Directive 2010/63/EU (European Commission, 2016).

Adult zebrafish were kept in standard-size tanks with a 3.5 L capacity with a maximum of 35 fish per tank (both males and females) and provided with a water recycling system. The zebrafish were fed twice a day and maintained in a controlled humidity (50–60%) and temperature (25°C). All the water parameters, including temperature, pH, salinity, and dissolved gases were constantly monitored to

be kept under the physiological range. The fish holding rooms were maintained under a day-night automatic cycle of 14 h light and 10 h dark.

For the *in vivo* experiments, adult zebrafish were crossed three days before the beginning of the experiment, into slopping breeding tanks that allow the eggs to fall and thus avoid them from being eaten by adult zebrafish. Aiming to enrich the environment and mimic zebrafish's natural habitat, synthetic algae were used in the breeding tanks. On the following morning, adult zebrafish were housed in their original tanks, while the embryos were harvested, with the help of strainers, to Petri dishes (approximately 50 embryos per dish) filled with E3 medium. The Petri dishes were posteriorly incubated at 28°C for the two following days.

The lines used for xenograft generation were Tg(fli1:EGFP) which allows the visualization of blood and lymphatic endothelial cells through the expression of EGFP under the control of fli1 promoter. Tg(mpeg1.1:LOXP-DsRedx-LOXP-GFP) which allows the visualization of macrophages through the expression of dsRed under the control of mpeg1 promoter. Tg(fli1:EGFP, gata1:RFP) which allows the visualization of blood and lymphatic endothelial cells through the expression of GFP under the control of fli1 promoter and erythrocytes/thrombocytes through the expression of RFP under the control of gata1 promoter. The Portuguese institutional organizations—ORBEA (Órgão de Bem-Estar e Ética Animal/Animal Welfare and Ethics Body) and DGAV (Direção Geral de Alimentação e Veterinária/Directorate General for Food and Veterinary) have approved this study and its corresponding protocols.

METHOD DETAILS

Patient sample processing for cryopreservation

Surgical resected samples were collected in sterile conditions during surgery, submerged in Dulbecco's Modified Eagle Medium: Nutrient Mixture F-12 (DMEM/F12; Life Technologies, ref. 12634010) at 4°C and transported to the laboratory (maximum within 1h post-surgery). Tissue samples were then cryopreserved in 90% (v/v) fetal bovine serum (FBS; Sigma-Aldrich, ref F7524) and 10% (v/v) DMSO (Thermo Fisher Scientific, ref. 1010348) until microinjection.

Malignant effusions (ascitic and pleural) were collected by the standard methods used in the Champalimaud Foundation medical center, into standard collection bags by routine paracentesis or thoracocentesis, and carried to the laboratory, at room temperature. The total volume of effusion was centrifuged at 300 xg, for 5 min at room temperature. The cell pellet was resuspended in 1x red blood cell lysis buffer (Abcam, ref ab204733) to remove erythrocytes. The cell pellets were then cryopreserved in 90% (v/v) fetal bovine serum (FBS; Sigma-Aldrich, ref F7524) and 10% (v/v) DMSO (Thermo Fisher Scientific, ref. 1010348) until microinjection.

Patient sample processing for zebrafish microinjection

Tissue was thawed in a water bath at 37°C, diluted in 40 mL of DPBS1X and centrifuged at 4°C, 300 xg for 5 min. The sample was then minced using a scalpel in the injection MIX (Table S2) and the obtained tissue fragments were filtered through a 70 μm cell strainer and labeled with a Deep Red (CellTracker, Thermo Fisher Scientific, ref C34565) at 1:1000 dilution (stock 10 μM), on ice. Staining was performed according to manufacturer's instructions. Tumor cells were then resuspended in the injection MIX (Table S2). Before microinjection, a small portion of the cell pellet was collected to perform cell smears in adhesion microscope slides (TOMO, Matsumani, ref TOM-1190). The cell smears were immediately fixed in cold methanol over night at 4°C, and preserved in Poly(ethylene glycol) (PEG) (Sigma-Aldrich, ref P2402) at room temperature [16] for further cell characterization, namely quantification of PAX8-positive cells, to guarantee a minimum of 10%.

Cells isolated from malignant effusions were thawed, centrifuged at 4°C, 300 xg, for 5 min and then resuspended in the injection MIX (Table S2). The cell suspension was filtered through a 70 μm cell strainer and labeled with a Deep Red (CellTracker, Thermo Fisher Scientific, ref C34565) at 1:1000 dilution (stock 10 μM), on ice. All the following steps were performed using the same protocol developed for cells isolated from tissue fragments.

Zebrafish patient-derived xenograft microinjection

Tumor cells were microinjected into the perivitelline space (PVS) of anesthetized 2 days post fertilization (dpf) zebrafish embryos [9,14,16]. Following microinjection, all embryos were maintained at 34°C until the end of the experiments in E3 medium. At 1day post-injection (dpi), zAvatars were screened regarding the presence or absence of a tumoral mass in the PVS. zAvatars without cells in the PVS, with severe edema or with cell debris were discarded. All xenografts were kept at 34°C until the end of the experiment.

Drug administration

At 1dpi zAvatars were randomly distributed to treatment groups: control (E3 medium) or selected chemotherapy. Treatments were administered for two consecutive days and replaced daily. The maximum tolerated concentration (MTC) of anti-cancer drugs was determined using, as a reference, the maximum patient's plasma concentration and testing different doses in non-injected embryos [17–20]. For all treatments, the highest dose without toxic effects was chosen (Table S3). Besides the addition to the E3 medium at 1dpi, when patients underwent treatment with bevacizumab monoclonal antibody, this was also added to the cell pellet prior to injection at 100 ng/mL [20]. At 3dpi, zAvatars were sacrificed with Tricaine 25x (Table S4, Sigma-Aldrich, ref. 886-86-2) and fixed with 4% (v/v) formaldehyde (FA) (ThermoFisher Scientific, ref. 28906) at 4°C overnight and preserved at −20°C in 100% (v/v) methanol (VWR Chemicals, ref. 20847.320) for long-term storage.

Whole-mount immunofluorescence

Whole-mount immunofluorescence was performed during three consecutive days. On day one zAvatars, previously stored in MeOH at -20°C, were submitted to a rehydration process through MeOH series: 100% (v/v) - 75% (v/v) - 50% (v/v) - 25% (v/v) and - 1X Phosphate Buffered Saline (PBS). If xenografts were not stored in MeOH, the rehydration process was not necessary. zAvatars were permeabilized by washing with PBS-Triton 0.1% (v/v) (Thermofisher Scientific, ref. 10254583) 4 times for 5 min, at room temperature. Samples were then washed with milliQ water (Merck Millipore, ref.: CDUFBI001) for 5 min and then incubated in 100% (v/v) acetone (Fisher Scientific, ref.: A/0606/17)) for 7 min at -20°C. Xenografts were washed with PBS-triton 0.1% (v/v) 2 times for 5 min and blocked with the blocking solution (Table S5) for 1 h at room temperature. zAvatars were then incubated with specific primary antibodies for 1 h at room temperature and then overnight at 4°C.

On the second day, zAvatars were washed with PBS-Triton 0.1% (v/v), 2 times for 10 min at room temperature, and then 4 times for 30 min. After washing, zAvatars were incubated with specific secondary antibodies diluted at 1:400 and DAPI (stock 5 mg/mL) diluted at 1:100, for 1 h at room temperature and then overnight at 4°C. From this point onwards and to preserve the fluorescence signal, samples were protected from light.

On the third day, zAvatars were washed with PBS-Tween 0.05% (v/v) (Fisher Scientific, ref BP337-100) 4 times for 15 min at room temperature, fixed with 4% (v/v) FA for 20 min at room temperature, and then washed again with PBS-Tween 0.05% (v/v) 2 times for 5 min at room temperature. The zAvatars were mounted in mowiol aqueous mounting media (Sigma-Aldrich, ref. 81381) in coverslips (Epredia, ref.: BB02400600AC13MNZ0) for confocal microscopy imaging and stored at 4°C on disposable cardboard slide trays. All antibodies used are described in Table S6.

Immunofluorescence in smears

PEG was removed with ethanol (EtOH) (Fisher Scientific, ref.: E/0650DF/C17) series (100% (v/v), 75% (v/v), 50% (v/v) and 25% (v/v)) for rehydration. For some primary antibodies (αSMA and CD5), antigen retrieval with Sodium Citrate Buffer 0.01 M with 0.05% (v/v) Tween 20, pH 6, was needed. The smears were submitted to permeabilization with PBS-Triton 0.1% (v/v). Smears that did not undergo antigen retrieval reached the permeabilization step immediately after PEG removal.

After permeabilization, the smears were surrounded with a hydrophobic pen, to avoid drying and incubated with the blocking solution (Table S5) for 30 min at room temperature After blocking, the smears were incubated with the primary antibodies (Table S6) for 2h at room temperature and then washed with PBS-triton 0.1% (v/v) 3 times for 10 min, and then incubated with the secondary antibody (Table S6), for 2h at room temperature. The smears were washed with PBS-triton 0.1% (v/v) 3 times for 10 min and incubated with DAPI (stock 5 mg/mL) (15:10000) (Sigma-Aldrich, ref. 10236276001) for 10 min at room temperature. The smears were washed with PBS-triton 0.1% (v/v) 3 times for 5 min and finally mounted in mowiol medium using a coverslip and stored at 4°C on disposable cardboard slide trays.

Imaging and quantification

Images of tumors in zAvatars were acquired in a Zeiss LSM 910 confocal microscope and BC43 Andor spinning disk microscope, with 5 μm interval between z-stacks. Images were analyzed using ImageJ software, using the Cell Counter plugin [21] for quantification of apoptotic bodies. The tumor size (sum of the total area of human mitochondria positive cells per slice) and percentage of activated caspase 3 were quantified manually by counting all cells in every slice of the tumor [15,22].

The implantation rate was quantified by calculating the number of zAvatars with a tumor at the end of the assay (3 dpi) divided by the total number of zAvatars at the end of the assay [15,22].

The metastatic burden was quantified by calculating the percentage of zAvatars with tumor cells detected at distant sites from the injection site, such as the head, eye, gills, or caudal hematopoietic region (CHT) at 3 dpi [15,22]. zAvatars with metastases in 2 or more spots were considered to have multiple-metastatic sites.

A SUM projection of the total activated caspase 3 with the region of interest (ROI) was performed in the analysis of the off-label options. The ROI was defined by the human mitochondria positive cells in each slice of the tumor.

QUANTIFICATION AND STATISTICAL ANALYSIS

Statistical analysis was performed using GraphPad Prism 9 and IBM SPSS software version 29.0.2. zAvatars numeric variables were challenged by normality tests (D'Agostino & Pearson and the Shapiro-Wilk). Data with assumed normal distribution were analyzed by independent *t* test. Variables with unknown distribution were analyzed by the Mann–Whitney test. $\chi 2$ test was performed to test associations with categorical variables. For metastases incidence analysis the Fisher's exact test was used. For all the statistical analysis, *p* value (p) is from a two-tailed test with a confidence interval of 95%. Statistical differences were considered significant whenever $p < 0.05$ and statistical output was represented by stars as follows: non-significant (ns) > 0.05, $*p \leq 0.05$, $**p \leq 0.01$, $***p \leq 0.001$, $****p \leq 0.0001$. Whenever a value suggestively deviating from the variables mean was observed, a comprehensive examination of the dataset was conducted using the "GraphPad Outlier" tool.

Receiver operating characteristic (ROC) analysis was performed using IBM SPSS software version 29.0.2 software, considering response to treatment (no-progression disease) as a positive event. Kaplan–Meier curves were performed using GraphPad Prism software and compared with the logrank test. We used the mean and the median as measures of central tendency. We computed the standard deviation and also calculated the min and max of variables, related to the patient that it means implicitly the amplitude of the variable. Since all graphs have an associated *p*-value, i.e., the existence of an inferential procedure, the variability was visualized by the SEM, resulting from the 95% confidence interval, i.e., the uncertainty about the population mean.

Cell Reports Medicine, Volume 7

Supplemental information

zAvatar-test—A functional precision model to personalize ovarian cancer treatments: Results from a co-clinical study

Marta F. Estrada, Filipa Amorim, Filipa Ferreira da Silva, Cátia Rebelo de Almeida, Márcia Fontes, Ricardo Coelho, Sónia Ferreira, Rita Canas-Marques, Mireia Castillo-Martin, João Casanova, Maria de Lurdes Batarda, Elisa Yaniz-Galende, Audrey LeFormal, Ana Marreiros, Francis Jacob, Viola Heinzelmann-Schwarz, Alexandra Leary, Henrique Nabais, and Rita Fior

Figure S1

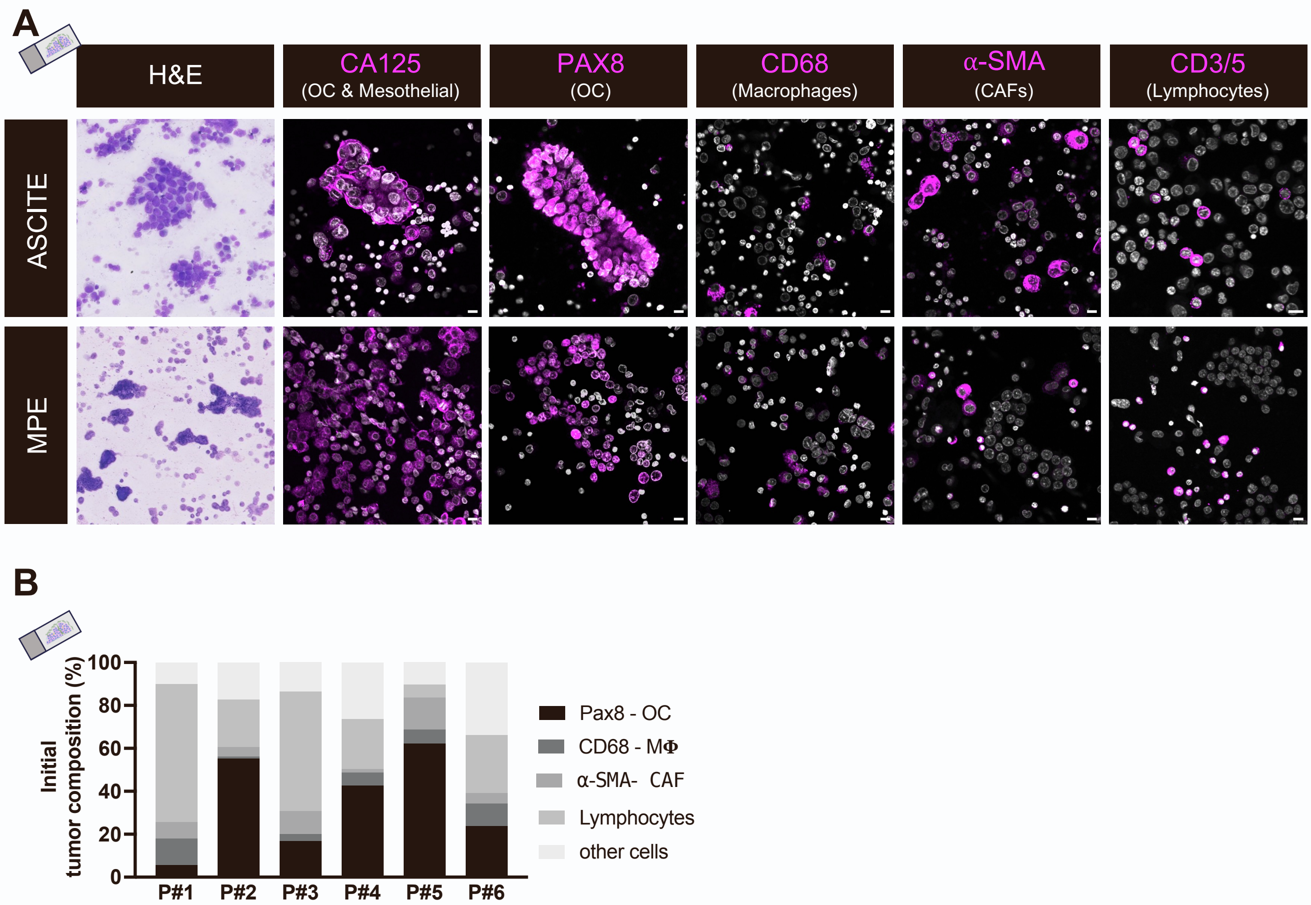

Figure S1. Comparison between samples obtained from different malignant effusions. Related to Figure 1. (A) Immunofluorescence characterization of patient cells before microinjection in smears, showing the presence of multiple cell types, including tumor cells (PAX8), tumor or mesothelial cells (CA125), macrophages (CD68), cancer-associated fibroblasts (a-SMA), lymphocytes (CD3/5), and erythrocytes (Giemsa staining). All immunofluorescence images are composites. **(B)** Analysis of cell composition in initial patient sample (smears) of the different cell populations.

Figure S2

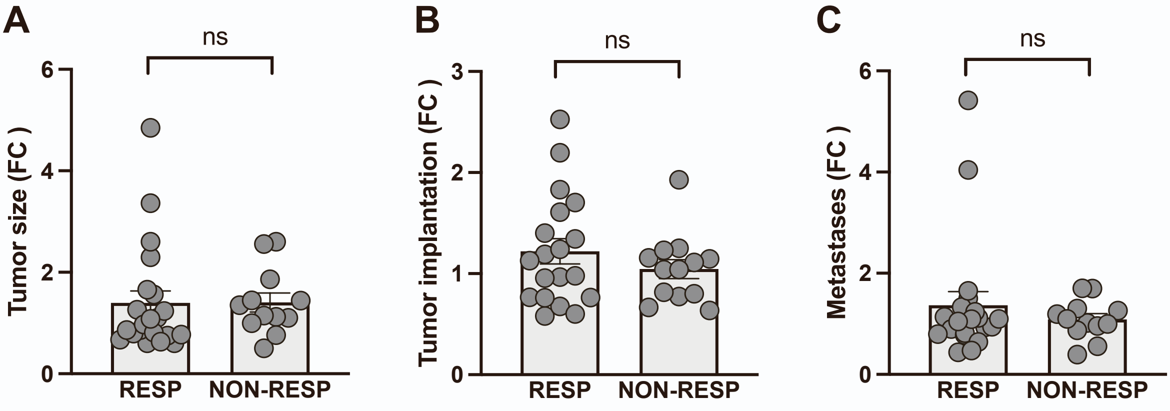

Figure S2. Tumor size, tumor implantation and micrometastases incidence in zAvatars in responder vs non-responder patients. Related to Figure 3. (A) Analysis of tumor size, **(B)** tumor implantation and **(C)** micrometastases incidence in zAvatars from responder vs non-responder patients. Data is expressed as AVG±SEM. Data were analyzed using unpaired two-sided Mann–Whitney test (ns > 0.05). Each dot represents one zAvatar. FC: fold change. RESP: responder. NON-RESP: non-responder.

Figure S3

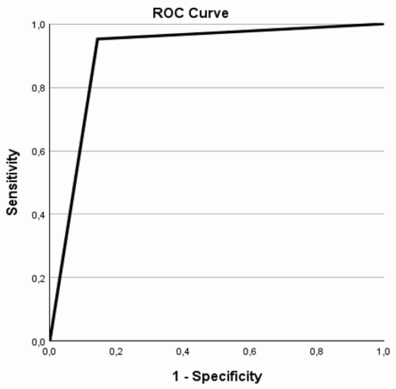

Figure S3. The zAvatar-test to discriminate responder from non-responder patients. Related to Figure 3. Receiver Operating Characteristic (ROC) curve analysis of the average fold change of apoptosis for both responder (N = 19 patients) and non-responder patients (N = 13 patients) of all 32 patients. The area under the curve was 0.905.

Figure S4

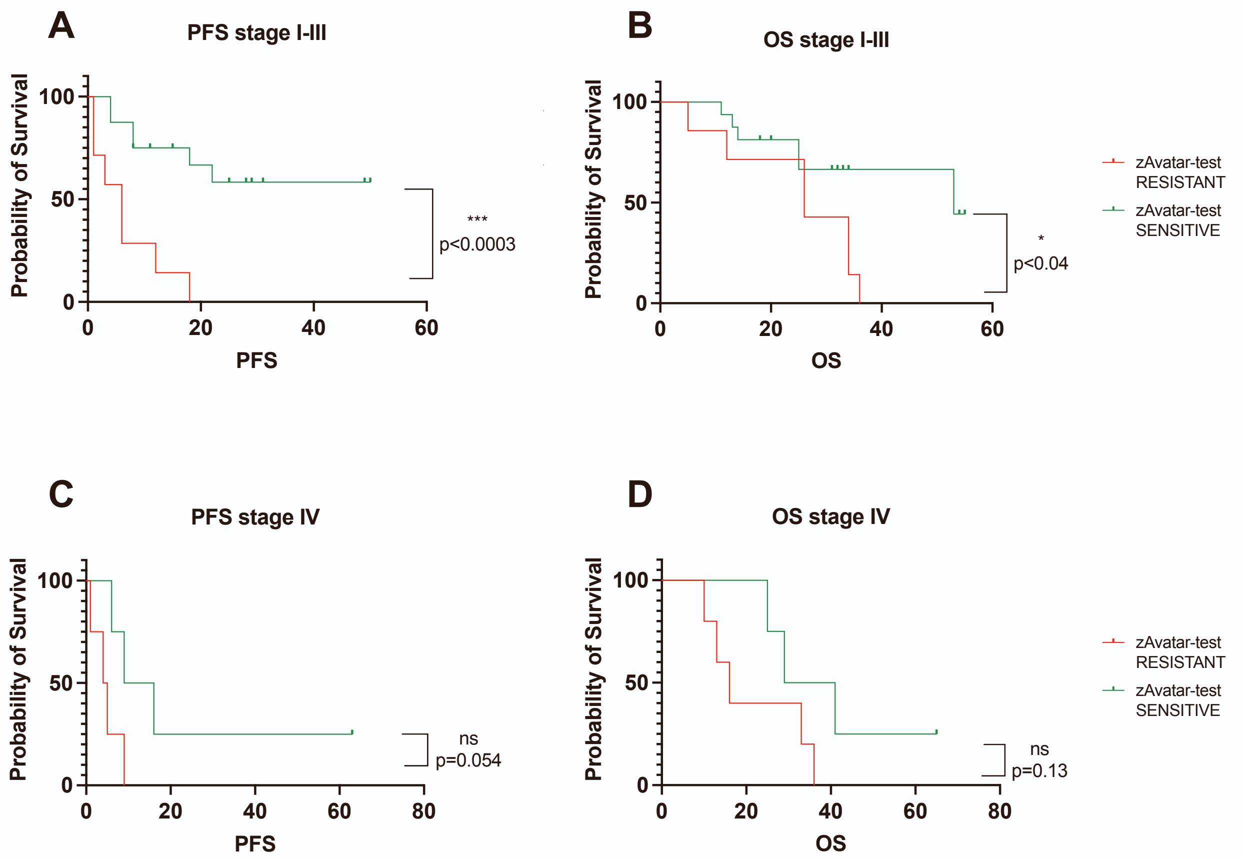

Figure S4. PFS and OS of patients with sensitive vs resistant zAvatar-test. Related to Figure 4. Kaplan–Meier survival curves to compare the PFS and OS of patients based on sensitivity or resistance of their zAvatar-test. Kaplan–Meier for PFS **(A, C)** and OS **(B, D)**. PFS: progression free survival. OS: overall survival.

Table S1. Study Population Baseline Characteristics. Related to Figure 1.

Study Population Baseline Characteristics (n=32)	
**Age at diagnosis - Years, average (s)**	59.4 (13.5)
**CA125 at sample collection - U/L, median (min-max)**	581 (30-15000)
**Histological types, n (%)**	
**Serous high grade**	28 (87.5%)
**Serous low grade**	2 (6.3%)
**Mucinous**	1 (3.1%)
**Clear Cell**	1 (3.1%)
**Neoadjuvant Chemotherapy, n (%)**	18 (56.3%)
**Primary surgery, n (%)**	29 (90.6%)
**FIGO staging, n (%)**	
**IA, n (%)**	2 (6.3%)
**IIIB, n (%)**	1 (3.1%)
**IIIC, n (%)**	20 (62.5%)
**IVA, n (%)**	4 (12.5%)
**IVB, n (%)**	5 (15.6%)
**BRCA mutation, n (%)**	2 (6.7%)
**Consolidation Chemotherapy, n (%)**	22 (68.8%)
**iPARP after consolidation CT, n (%)**	14 (43.8%)
**Bevacizumab after consolidation CT, n (%)**	5 (15.6%)
**Ascites at sample collection, n (%)**	16 (50%)
**Pleural effusion at sample collection, n (%)**	6 (18.8%)
**Ascites and pleural effusion at sample collection, n (%)**	1 (3.1%)

Table S2. Reagents for MIX1 preparation. Related to STAR Methods.

Reagents	Concentration	Vendor	Cat.no
Advanced DMEM/F12		ThermoFisher Scientific	12634010
Hepes	1%	ThermoFisher Scientific	15630106
MEM Non-Essential Amino Acids Solution	1x	ThermoFisher Scientific	1114005
B-27™ Supplement	1x	ThermoFisher Scientific	17504044
Putrescine dihydrochloride	10 μg/mL	Sigma-Aldrich	P5780
Nicotinamide	10 mM	Sigma-Aldrich	72340
N-acetylcysteine	1.25 mM	Sigma-Aldrich	A9165
Hydrocortisone	0.5 μg/mL	Sigma-Aldrich	H0888
glutaGRO Supplement	1x	Corning	MT25015CI
Insulin-Transferrin-Selenium	1% (v/v)	Corning	15383661
Y-27632	10 μM	Selleckchem	S6390
EGF	50 ng/mL	Peprotech	AF-100-15
Primocin	100 μg/mL	Invivogen	ant-pm-1
Universal Nuclease	37.5 U/mL	Pierce	88700

Table S3 – Treatments administered to zAvatars. Related to STAR Methods.

Scheme	Generic name	Cmax Patients (μM)	Concentration in zAvatar medium (μM)	Reference for Cmax Patients
**carboplatin + paclitaxel**	carboplatin	135	675	[S1]
	paclitaxel	4.27	0.34	[S1]
**carboplatin + paclitaxel + olaparib**	carboplatin	135	675	[S1]
	paclitaxel	4.27	0.34	[S1]
	plaparib	5	50	[S1]
**carboplatin + paclitaxel + niraparib**	carboplatin	135	675	[S1]
	paclitaxel	4.27	0.34	[S1]
	niraparib	0.00165	250	[S1]
**carboplatin + paclitaxel + bevacizumab**	carboplatin	135	675	[S1]
	paclitaxel	4.27	0.34	[S1]
	bevacizumab	0.6-0.9	1.68	[S2]
**carboplatin + gemcitabine**	carboplatin	135	675	[S1]
	gemcitabine	89.3	160	[S1]
**carboplatin + doxorubicin**	carboplatin	135	675	[S1]
	doxorubicin	6.73	1.2	[S1]
**paclitaxel**	paclitaxel	4.27	0.34	[S1]
**doxorubicin**	doxorubicin	6.73	1.2	[S1]
**topotecan**	topotecan	0.02	0.02	[S1]
**paclitaxel + bevacizumab**	paclitaxel	4.27	0.34	[S1]
	bevacizumab	0.6-0.9	1.68	[S2]
**doxorubicin + bevacizumab**	doxorubicin	6.73	1.2	[S1]
	bevacizumab	0.6-0.9	1.68	[S2]
**topotecan + bevacizumab**	topotecan	0.02	0.02	[S1]
	bevacizumab	0.6-0.9	1.68	[S2]
**venetoclax**	venetoclax	4.48	0.448	[S1]
**carboplatin + paclitaxel + venetoclax**	carboplatin	135	675	[S1]
	paclitaxel	4.27	0.34	[S1]
	venetoclax	4.48	0.448	[S1]

Table S4 – Composition of the Tricaine solution. Related to STAR Methods.

Tricaine 25x	Quantity
Tricaine powder	2 g
Reverse osmosis water	500 mL
1 M Tris (pH 9)	10 mL
Adjust to pH 7	250 µL

Table S5 – Composition of the blocking solution. Related to STAR Methods.

Reagent	Quantity
PBS (1x)	To a final volume of 50 mL
BSA	0.5 g
DMSO	500 µL
Triton 10% (w/v)	250 µL
Goat serum	750 µL

Table S6 – Antibodies used in immunofluorescent staining. Related to STAR Methods.

	Antibody	Supplier	Host animal	Dilution factor	Clones
**Primary antibodies**	Anti-Human mitochondria	Sigma Life Sciences	Mouse	1:50	113- 1
	Anti-Caspase 3	Cell Signaling Technologies	Rabbit	1:100	5A1E
	Anti-PAX8	Abcam	Rabbit	1:500	EPR18715
	Anti-CA125	Leica Biosystems	Mouse	Ready to use	OV185:1
	Anti-CD68	Leica Biosystems	Mouse	Ready to use	514H12
	Anti-aSMA	Abcam	Rabbit	1:2500	EPR5368
	Anti-CD3	Leica Biosystems	Mouse	Ready to use	CD3-565-L-CE
	Anti-CD5	Leica Biosystems	Mouse	Ready to use	CD5-4C7-L-CE
**Secondary antibodies**	Anti-mouse 488	ThermoFisher Scientific	Goat	1:400 (whole mount IF) 1:200 (smear)	
	Anti-mouse 594	ThermoFisher Scientific	Goat	1:400 (whole mount IF) 1:200 (smear)	-
	Anti-rabbit 488	ThermoFisher Scientific	Goat	1:400 (whole mount IF) 1:200 (smear)	-
	Anti-rabbit 594	ThermoFisher Scientific	Goat	1:400 (whole mount IF) 1:200 (smear)	-
	DAPI	Invitrogen	-	1:100 (whole mount IF) 15:10000 (smear)	-

Supplemental references

[S1] D.R. Liston, M. Davis, Clinically Relevant Concentrations of Anticancer Drugs: A Guide for Nonclinical Studies, Clin. Cancer Res. 23 (2017) 3489–3498. doi:10.1158/1078-0432.CCR-16-3083.

[S2] C. Rebelo de Almeida, R.V. Mendes, A. Pezzarossa, J. Gago, C. Carvalho, A. Alves, et al., Zebrafish xenografts as a fast screening platform for bevacizumab cancer therapy, Commun Biol. 3 (2020) 299–13. doi:10.1038/s42003-020-1015-0.
